# Integrating Precision Nutrition with GLP-1 Receptor Agonist Therapy: Mechanisms, Clinical Outcomes, and Pharmacoeconomic Implications

**DOI:** 10.3390/ph19081230

**Published:** 2026-08-05

**Authors:** Stefano Ruga, Elisa Matarese, Roberto Bava, Carmen Lombardi, Tiziana Dimatteo, Leonardo Miscio, Massimo Raponi, Antonio Giordano, Clementina Sansone, Giovanna Liguori, Renato Lombardi

**Affiliations:** 1 Department of Health Sciences, Magna Graecia University of Catanzaro, 88100 Catanzaro, Italy; rugast1@gmail.com; 2 Vita-Salute San Raffaele University, 20132 Milan, Italy; e.matarese@studenti.unisr.it; 3 Degree Programme in Medicine and Surgery, Catholic University of the Sacred Heart, 00168 Rome, Italy; 4 Local Health Authority of Foggia (ASL Foggia), 71121 Foggia, Italygiovanna.liguori@aslfg.it (G.L.); renato.lombardi@aslfg.it (R.L.); 5 Sbarro Institute for Cancer Research and Molecular Medicine, Center for Biotechnology, Department of Biology, College of Science and Technology, Temple University, Philadelphia, PA 19122, USA; 6 Department of Medical Biotechnology, University of Siena, 53100 Siena, Italy; 7 Anton Dohrn Zoological Station, Villa Comunale, 80121 Naples, Italy; clementina.sansone@szn.it; 8 Sbarro Health Research Organization ETS, 10060 Candiolo, Italy

**Keywords:** personalized medicine, gut microbiota, drug–nutrient interactions, body composition, digital health, cost-effectiveness, obesity management, Foods for Special Medical Purposes (FSMPs)

## Abstract

The integration of precision nutrition with pharmacological therapies has been proposed as a strategy to optimize therapeutic efficacy, tolerability, and patient-centered outcomes in chronic metabolic diseases. Glucagon-like peptide-1 receptor agonists (GLP-1 RAs) have substantially advanced the treatment of obesity and type 2 diabetes mellitus by promoting substantial weight loss and improving glycemic control and cardiometabolic risk profiles. However, significant interindividual variability in therapeutic response, frequent gastrointestinal adverse events leading to treatment discontinuation, and the potential loss of lean body mass remain major clinical challenges that limit real-world therapeutic efficiency. Emerging evidence suggests that nutritional status, dietary patterns, body composition, inflammatory burden, and gut microbiota composition may influence both the efficacy and tolerability of GLP-1-based therapies. This review explores the role of precision nutrition as an adjunctive strategy to support GLP-1 RA treatment outcomes through personalized dietary interventions and targeted nutritional support. Particular attention is given to protein intake optimization for lean mass preservation, micronutrient adequacy, microbiota modulation, anti-inflammatory dietary approaches, and the management of gastrointestinal symptoms. Furthermore, the review explores the emerging contribution of nutrigenomics, metabolomics, microbiome-based stratification, digital health technologies, and artificial intelligence in advancing pharmacometabolic personalization. The potential pharmacoeconomic implications of integrated nutritional–pharmacological strategies are discussed, including the potential for improved adherence to reduce treatment-related costs, prevent complications, and enhance long-term metabolic sustainability. While current evidence supports the mechanistic rationale for combining precision nutrition with GLP-1 receptor agonist therapy, it must be emphasized that direct evidence from prospective clinical trials evaluating such integrated strategies is limited. Most recommendations are based on indirect evidence from general weight-loss or metabolic disease populations, and their efficacy, specifically in GLP-1 RA-treated patients, remains to be demonstrated.

## 1. Introduction

Obesity and type 2 diabetes mellitus (T2DM) represent major global health challenges, closely associated with increased cardiovascular risk, chronic low-grade inflammation, metabolic dysfunction, and reduced life expectancy. Over the past decades, the prevalence of obesity has risen dramatically worldwide, contributing substantially to the burden of insulin resistance, metabolic syndrome, cardiovascular disease, chronic kidney disease, and metabolic dysfunction-associated steatotic liver disease (MASLD). According to the World Health Organization, global obesity rates have nearly tripled since 1975, with more than one billion individuals currently living with obesity worldwide [1]. The recognition of obesity as a chronic relapsing disease requiring long-term multidisciplinary management has reshaped therapeutic paradigms and highlighted the need for integrated pharmacological and non-pharmacological strategies to achieve sustained metabolic health improvements [2].

The conceptualization of obesity as a disease state requiring medical intervention, rather than a consequence of lifestyle choices alone, has been foundational to the development of pharmacological treatment paradigms [3].

In this context, glucagon-like peptide-1 receptor agonists (GLP-1 RAs) have emerged as one of the most significant pharmacological advances in the treatment of obesity and T2DM. These agents improve glycemic control and promote weight loss through multiple mechanisms, including the enhancement of glucose-dependent insulin secretion, suppression of glucagon secretion, delayed gastric emptying, and modulation of appetite and satiety pathways within the central nervous system [4]. Beyond glycemic regulation, GLP-1 RAs exert beneficial cardiovascular and renal effects, leading to their increasing use in cardiometabolic medicine [5,6].

The scope of GLP-1 RA applications continues to expand, with recent trials demonstrating efficacy across a spectrum of obesity-related conditions, including heart failure with preserved ejection fraction, chronic kidney disease, and metabolic dysfunction-associated steatotic liver disease [7,8].

Large randomized controlled trials have demonstrated the remarkable efficacy of GLP-1-based therapies in obesity management: the STEP-1 trial reported that once-weekly semaglutide 2.4 mg combined with lifestyle intervention induced a mean weight reduction of 14.9% at 68 weeks compared with 2.4% in the placebo group [9], while the SURMOUNT-1 trial demonstrated that tirzepatide, a dual glucose-dependent insulinotropic polypeptide (GIP)/GLP-1 receptor agonist, produced body weight reductions of up to 20.9% in adults with obesity, establishing a new benchmark for pharmacological obesity treatment [10].

Subsequent trials have extended these findings to diverse populations and higher-dose regimens. The STEP 7 trial confirmed the efficacy and safety of semaglutide 2.4 mg in a predominantly East Asian population with overweight or obesity [11], while the STEP UP T2D trial demonstrated that semaglutide 7.2 mg produced superior weight loss compared to the standard 2.4 mg dose in adults with obesity and type 2 diabetes [12]. The SURPASS-CVOT trial is further evaluating cardiovascular outcomes with the dual GIP/GLP-1 receptor agonist tirzepatide compared to dulaglutide, reflecting the ongoing commitment to establishing the cardioprotective benefits of dual incretin agonists [13].

Furthermore, evidence from the SELECT trial highlighted the cardiovascular benefits of semaglutide in patients with obesity and established cardiovascular disease, showing a significant reduction in major adverse cardiovascular events [14].

Despite these remarkable therapeutic advances, several clinically relevant limitations remain that constrain real-world therapeutic efficiency. Considerable interindividual variability in treatment response has been consistently observed, with some patients achieving profound metabolic improvements while others experience suboptimal weight loss or reduced tolerability [15]. Gastrointestinal adverse events, including nausea, vomiting, constipation, diarrhea, and delayed gastric emptying, remain among the most common causes of treatment discontinuation and impaired adherence, with dose-dependent severity significantly impacting long-term treatment sustainability in real-world settings [16,17].

The importance of treatment persistence is underscored by the STEP 4 trial, which demonstrated that continuation of semaglutide is necessary to maintain weight loss, as patients randomized to placebo after an initial 20-week run-in regained a substantial proportion of lost weight [18].

Another emerging concern relates to the potential loss of lean body mass during rapid weight reduction induced by GLP-1-based therapies, particularly relevant in older adults, frail individuals, and patients with sarcopenic obesity, where excessive lean mass reduction may negatively affect physical function, resting energy expenditure, and long-term metabolic health [19,20]. Clinical practice guidelines have increasingly emphasized the importance of individualized, multidisciplinary approaches to obesity management that integrate pharmacological, nutritional, and behavioral interventions [21].

Emerging evidence suggests that nutritional status, dietary composition, inflammatory burden, and gut microbiota profiles may substantially influence both the efficacy and tolerability of GLP-1 receptor agonists. Gut microbiota-derived metabolites, including short-chain fatty acids, modulate endogenous GLP-1 secretion, insulin sensitivity, and systemic inflammation, providing a mechanistic basis for microbiota-targeted nutritional interventions [22,23]. In parallel, advances in nutrigenomics, metabolomics, and digital health technologies are increasingly enabling the development of individualized nutritional approaches tailored to specific metabolic phenotypes and therapeutic needs [24]. These advances enable a paradigm shift from standardized lifestyle recommendations to precision nutrition strategies that account for individual variability in drug response, nutritional needs, and metabolic adaptation, with potential implications for therapeutic efficiency and healthcare sustainability.

Within this evolving framework, the present review aims to provide a comprehensive overview of the role of precision nutrition as an integrative strategy to optimize the therapeutic efficiency of GLP-1 receptor agonists. The review examines the mechanisms underlying interindividual variability in treatment response, nutritional challenges arising during GLP-1 therapy, and precision nutrition strategies for body composition preservation, microbiota modulation, and the management of gastrointestinal adverse events. Furthermore, it explores the contribution of biomarkers, multi-omics approaches, digital health technologies, and artificial intelligence to pharmacometabolic personalization. Finally, the pharmacoeconomic implications of integrated nutritional–pharmacological strategies are discussed, including the potential for improved adherence and reduced treatment-related complications to enhance cost-effectiveness and support sustainable obesity management.

**Figure 1:**
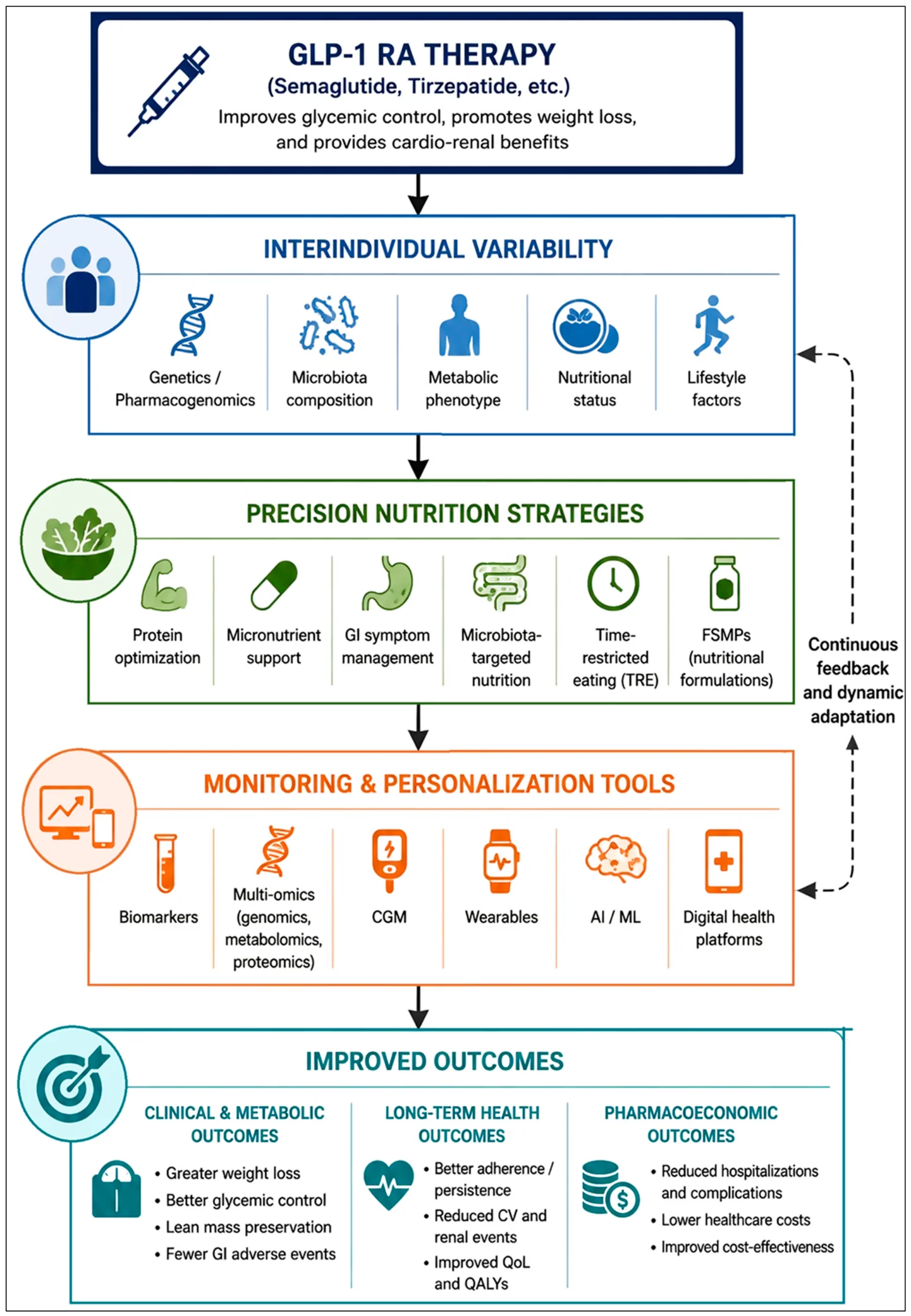
Proposed integrative framework for precision nutrition optimization of GLP-1 receptor agonist therapy. Interindividual variability arising from genetic, metabolic, microbiome, nutritional, and lifestyle factors influences the efficacy and tolerability of GLP-1 receptor agonist (GLP-1 RA) therapy. Precision nutrition strategies, supported by biomarkers, multi-omics approaches, and digital health technologies, may enable dynamic treatment personalization. The clinical and metabolic outcomes depicted in the lower panel have been demonstrated in GLP-1 RA trials; however, the specific contribution of precision nutrition interventions to these outcomes has not been isolated in prospective studies and represents a hypothesis requiring validation. Pharmacoeconomic outcomes and the continuous feedback loop similarly represent hypothesized benefits for which direct evidence is currently lacking. Abbreviations: AI/ML, Artificial Intelligence/Machine Learning; CGM, Continuous Glucose Monitoring; CV, Cardiovascular; FSMPs, Foods for Special Medical Purposes; GI, Gastrointestinal; GLP-1 RA, Glucagon-Like Peptide-1 Receptor Agonist; QALY, Quality-Adjusted Life Year; QoL, Quality of Life; TRE, Time-Restricted Eating.

The conceptual framework for integrating precision nutrition with GLP-1 receptor agonist therapy is illustrated in Figure 1.

## 2. Methods

This review was conducted as a narrative synthesis of the literature at the intersection of precision nutrition and glucagon-like peptide-1 (GLP-1) receptor agonist therapy. The aim was to provide a comprehensive overview of mechanistic rationale, clinical evidence, and emerging research directions, rather than to perform a systematic review or meta-analysis.

### 2.1. Search Strategy and Selection Criteria

Relevant English-language articles published up to May 2026 were identified through searches of PubMed, Scopus, and Semantic Scholar. Search terms included combinations of the following keywords: “GLP-1 receptor agonist”, “semaglutide”, “liraglutide”, “tirzepatide”, “incretin”, “precision nutrition”, “personalized nutrition”, “dietary intervention”, “protein intake”, “body composition”, “lean body mass”, “sarcopenic obesity”, “gut microbiota”, “microbiome”, “short-chain fatty acids”, “gastrointestinal tolerability”, “micronutrient deficiency”, “Mediterranean diet”, “time-restricted eating”, “chrononutrition”, “polyphenols”, “omega-3 fatty acids”, “nutrigenomics”, “metabolomics”, “digital health”, “continuous glucose monitoring”, “artificial intelligence”, “pharmacoeconomics”, “cost-effectiveness”, “Foods for Special Medical Purposes”, and “FSMPs”. Boolean operators (AND, OR) were used to combine search terms. Searches were conducted between January and May 2026, with no restrictions on publication date; however, priority was given to articles published within the last 10 years (2016–2026) for clinical evidence.

After the removal of duplicates, titles and abstracts were screened for relevance by two authors, and full-text articles were retrieved for all potentially eligible records. Disagreements regarding inclusion were resolved through discussion with a third author. Additional articles were identified through citation tracking of key references and through the authors’ personal libraries.

Study selection prioritized: (a) randomized controlled trials and prospective observational studies conducted specifically in patients receiving GLP-1-based therapies; (b) systematic reviews and meta-analyses of nutritional interventions in obesity and type 2 diabetes; (c) mechanistic studies elucidating diet–drug–microbiome interactions relevant to incretin biology; and (d) health economic evaluations of obesity pharmacotherapy. Reference lists of the included articles were screened to identify additional relevant publications. Given the narrative nature of this review, formal quality assessment tools were not applied, and the selection and synthesis of evidence may reflect author judgment.

### 2.2. Evidence Classification

Throughout this review, evidence is classified to distinguish direct evidence obtained specifically in GLP-1 receptor agonist-treated populations from indirect evidence extrapolated from general weight-loss, nutrition, microbiome, or digital-health studies. In the summary tables, evidence is categorized as: (A) direct evidence from studies conducted in patients receiving GLP-1-based therapies; (B) indirect clinical evidence from weight-loss or metabolic disease populations not specifically receiving GLP-1 RAs; (C) mechanistic or preclinical evidence; and (D) expert opinion or hypothesis. Where recommendations are based primarily on indirect evidence, this is explicitly stated in the text.

### 2.3. Terminology

Throughout this review, the term “GLP-1 receptor agonists” (GLP-1 RAs) refers to selective glucagon-like peptide-1 receptor agonists (e.g., semaglutide, liraglutide, dulaglutide). Tirzepatide, a dual glucose-dependent insulinotropic polypeptide (GIP)/GLP-1 receptor agonist, is identified as such at each occurrence to distinguish it from selective GLP-1 RAs. The broader term “GLP-1-based therapies” is used when referring collectively to both selective GLP-1 RAs and dual incretin agonists. “Precision nutrition” is defined operationally in Section 6.1 to distinguish it from routine dietary advice.

## 3. Mechanisms of GLP-1 Receptor Agonists: Pharmacological Basis for Nutritional Integration

Glucagon-like peptide-1 receptor agonists (GLP-1 RAs) represent a major pharmacological advancement in the management of obesity and type 2 diabetes mellitus (T2DM), exerting pleiotropic effects on glucose homeostasis, energy balance, and cardiometabolic risk through a complex network of central and peripheral mechanisms. The GLP-1 receptor is a class B G protein-coupled receptor widely expressed in pancreatic islets, the gastrointestinal tract, vagal afferents, and multiple regions of the central nervous system involved in metabolic regulation. Activation of this receptor triggers intracellular signaling cascades primarily mediated by cyclic AMP (cAMP), protein kinase A (PKA), and exchange protein directly activated by cAMP 2 (Epac2), ultimately enhancing glucose-dependent insulin secretion and modulating multiple metabolic pathways [25,26]. The physiological basis for incretin-based chronic weight management has been comprehensively reviewed, emphasizing the integrated actions of GLP-1 on multiple organ systems [27].

Understanding these mechanisms is essential to appreciate how nutritional factors may interact with, and potentially amplify or attenuate, GLP-1 receptor signaling, a concept central to the integration of precision nutrition with incretin-based pharmacotherapy.

### 3.1. Central Regulation of Appetite, Energy Intake, and Hedonic Feeding Behavior

One of the most clinically relevant mechanisms of GLP-1 receptor agonists is the modulation of appetite and energy intake through central nervous system pathways. GLP-1 receptors are expressed in hypothalamic nuclei critical for energy homeostasis, including the arcuate nucleus, paraventricular nucleus, and nucleus tractus solitarius. Activation of these regions stimulates anorexigenic pro-opiomelanocortin (POMC) neurons while inhibiting orexigenic neuropeptide Y/agouti-related peptide (NPY/AgRP) neurons, leading to reduced hunger and increased satiety [28]. Beyond homeostatic regulation, GLP-1 RAs also influence hedonic feeding behavior by modulating mesolimbic reward circuits, including the ventral tegmental area and nucleus accumbens, reducing the motivational salience of highly palatable, energy-dense foods. Functional neuroimaging studies have demonstrated that GLP-1 receptor activation attenuates brain responses to food-related cues in reward-associated regions, supporting a central mechanism for sustained weight loss independent of gastrointestinal effects [29,30].

Clinically, these central effects translate into a sustained reduction in energy intake and progressive weight loss, as consistently demonstrated in large randomized controlled trials such as STEP-1 and SURMOUNT-1 [9,10]. Importantly, the shift in food preferences away from highly palatable, energy-dense foods and toward less calorically dense options has direct implications for nutritional management during GLP-1 therapy. While reduced preference for unhealthy foods is therapeutically desirable, it may also lead to reduced overall dietary variety and, if not guided, to inadequate intake of protein-rich and micronutrient-dense foods. This creates a clinical imperative for structured nutritional support that preserves diet quality within a reduced energy intake framework.

### 3.2. Regulation of Gastric Emptying and Gastrointestinal Function

GLP-1 receptor agonists exert significant effects on gastrointestinal motility, particularly through the delay of gastric emptying. This mechanism contributes to reduced postprandial glucose excursions by slowing nutrient delivery to the small intestine and enhances satiety through prolonged gastric distension and vagal afferent signaling. The effect is mediated via vagal pathways and modulation of enteric nervous system activity, with endogenous GLP-1 playing a physiological role in the regulation of antro-pyloro-duodenal motility [31].

However, these gastrointestinal actions are closely related to the most commonly reported adverse effects of GLP-1 receptor agonists, including nausea, vomiting, early satiety, bloating, and constipation. Such symptoms are dose-dependent, particularly pronounced during dose escalation phases, and represent a major cause of treatment discontinuation and reduced adherence in clinical practice [4,16]. It should be noted that with chronic GLP-1 RA administration, tachyphylaxis to the gastric emptying delay may occur, whereas central appetite-suppressing effects are maintained, suggesting that central mechanisms predominate in long-term weight regulation. In patients receiving GLP-1 RAs in combination with insulin or sulfonylureas, clinicians should be aware that severe vomiting or prolonged reductions in oral intake may precipitate hypoglycemia, particularly if glucose-lowering medications are not appropriately adjusted. Temporary reduction in insulin or sulfonylurea doses should be considered during acute gastrointestinal illness, and patients should be counseled on hypoglycemia recognition and management [16,17].

From a nutritional perspective, the delayed gastric emptying and associated gastrointestinal symptoms create specific challenges that can be addressed through dietary modification. Meal size reduction, lower fat intake to accelerate gastric emptying, modified food textures, and increased meal frequency represent evidence-based strategies to improve gastrointestinal tolerability without compromising the therapeutic benefits of delayed gastric emptying on postprandial glycemia. These dietary adaptations represent a key intersection between GLP-1 pharmacology and precision nutrition.

### 3.3. Insulin Secretion, Glucagon Suppression, and Islet Cell Function

A central mechanism of GLP-1 receptor agonists is the enhancement of glucose-dependent insulin secretion from pancreatic β-cells and the suppression of inappropriate glucagon release from pancreatic α-cells. This dual action improves both postprandial and fasting glycemic control while minimizing the risk of hypoglycemia, a distinguishing feature from other insulin secretagogues such as sulfonylureas. At the cellular level, GLP-1 receptor activation increases intracellular cAMP concentrations, which subsequently activate the PKA and Epac2 signaling pathways, facilitating insulin granule exocytosis, enhancing β-cell responsiveness to glucose, and promoting proinsulin biosynthesis [25,26]. Beyond these acute insulinotropic effects, GLP-1 signaling may support β-cell survival and function through anti-apoptotic pathways, suggesting potential disease-modifying properties in T2DM.

The glucose-dependent nature of GLP-1-mediated insulin secretion has important nutritional implications. Dietary patterns that produce more gradual postprandial glucose excursions, such as those rich in fiber, with low glycemic index, and with appropriate macronutrient distribution, may optimize the therapeutic effect of GLP-1 RAs by providing a more sustained and predictable glycemic stimulus for insulin secretion. Conversely, diets high in rapidly-absorbable carbohydrates may produce exaggerated postprandial glucose peaks that are more difficult to control pharmacologically, potentially requiring higher GLP-1 RA doses and increasing the risk of gastrointestinal side effects. This concept supports the rationale for aligning dietary composition with the pharmacodynamic profile of GLP-1 RAs [22,32].

### 3.4. Anti-Inflammatory and Cardiometabolic Effects

In addition to their metabolic actions, GLP-1 receptor agonists exert pleiotropic anti-inflammatory and cardioprotective effects that contribute to their clinical benefits beyond weight loss and glycemic control. Experimental and clinical studies have shown that GLP-1 signaling reduces systemic inflammation through the inhibition of nuclear factor-kappa B (NF-κB) activation, downregulation of pro-inflammatory cytokines such as tumor necrosis factor-alpha (TNF-α) and interleukin-6 (IL-6), and attenuation of oxidative stress pathways [33]. Furthermore, GLP-1 receptor activation has been associated with improved endothelial function, reduced vascular inflammation, and modulation of innate immune responses, including inhibition of the NLRP3 inflammasome, linking incretin signaling to the broader network of metabolic inflammation [25].

These anti-inflammatory effects are particularly relevant in the context of obesity, where chronic low-grade inflammation, driven by adipocyte hypertrophy, macrophage infiltration of adipose tissue, and gut-derived metabolic endotoxemia, represents a central pathophysiological mechanism linking excess adiposity to insulin resistance, cardiovascular disease, and metabolic dysfunction. Consistent with these mechanistic findings, the SELECT trial demonstrated that semaglutide significantly reduces major adverse cardiovascular events in patients with obesity and established cardiovascular disease, independent of its effects on glycemic control [14].

The convergence of GLP-1-mediated anti-inflammatory pathways with the well-established anti-inflammatory effects of specific dietary patterns, particularly the Mediterranean diet, rich in polyphenols, monounsaturated fats, and omega-3 fatty acids, suggests the potential for pharmacometabolic synergy. Dietary interventions that reduce systemic inflammatory tone may amplify the anti-inflammatory actions of GLP-1 RAs, while GLP-1-induced improvements in metabolic health may enhance the physiological response to healthy dietary patterns. This bidirectional relationship provides a mechanistic foundation for integrating anti-inflammatory nutritional strategies with GLP-1-based pharmacotherapy.

### 3.5. Nutritional Modulation of Endogenous Incretin Tone: Implications for Pharmacological Therapy

Beyond the pharmacological actions of exogenous GLP-1 receptor agonists, the endogenous incretin system is subject to significant nutritional modulation, a concept with direct implications for precision nutrition approaches during GLP-1 RA therapy. Enteroendocrine L cells, located primarily in the distal ileum and colon, secrete endogenous GLP-1 in response to nutrient ingestion through both direct luminal sensing mechanisms and indirect signals mediated by the enteric nervous system and circulating factors.

Specific dietary components have been identified as potent stimulators of endogenous GLP-1 secretion. Short-chain fatty acids (SCFAs), acetate, propionate, and butyrate, produced through bacterial fermentation of dietary fiber, stimulate L-cell GLP-1 release via activation of G protein-coupled receptors GPR41 and GPR43 [34]. Dietary proteins and amino acids, particularly glutamine and arginine, also stimulate GLP-1 secretion through calcium-sensing receptor activation and electrogenic uptake mechanisms. Furthermore, specific polyphenols, including those found in extra-virgin olive oil, berries, and tea, have been shown to modulate GLP-1 secretion through interactions with bitter taste receptors on L cells.

The concept of “incretin tone”, the basal and postprandial level of endogenous GLP-1 activity, is therefore modifiable through dietary composition. A diet rich in fermentable fiber, high-quality protein, and polyphenols may maintain a higher basal incretin tone, potentially enhancing the therapeutic effects of exogenous GLP-1 receptor agonists or allowing for effective treatment at lower pharmacological doses. Conversely, a diet low in fiber and high in ultra-processed foods may result in reduced endogenous GLP-1 tone, potentially necessitating higher pharmacological doses and increasing the risk of dose-dependent gastrointestinal adverse effects. This nutritional modulation of the endogenous incretin system supports a mechanistic basis for integrating precision nutrition with GLP-1 RA pharmacotherapy and suggests the hypothesis that dietary optimization may support endogenous incretin secretion in a manner that could complement exogenous GLP-1 receptor activation. This concept, sometimes referred to as dietary support of endogenous incretin tone, remains to be validated in clinical trials of GLP-1 RA-treated patients.

The pharmacological mechanisms of GLP-1 RAs, their clinical effects, and the corresponding nutritional implications are summarized in Table 1.

## 4. Interindividual Variability in GLP-1 Receptor Agonist Response: Implications for Personalized Nutritional Strategies

Despite the robust efficacy of glucagon-like peptide-1 receptor agonists (GLP-1 RAs) demonstrated across randomized controlled trials, real-world clinical outcomes reveal substantial interindividual variability in weight loss, glycemic response, and treatment tolerability. While some patients achieve profound and sustained metabolic improvements, others exhibit attenuated responses or early discontinuation due to limited efficacy or intolerable adverse effects. This heterogeneity is a fundamental challenge to the optimization of GLP-1-based therapies and provides a compelling rationale for personalized approaches that extend beyond standardized dosing regimens. Variability in GLP-1 RA response reflects a complex interplay between genetic predisposition, sex-specific biology, neuroendocrine regulation, gut microbiota composition, inflammatory status, body composition, and metabolic phenotype, factors that also influence nutritional requirements and dietary response [15,35]. Understanding the biological determinants of this variability is essential for developing precision nutrition strategies that can be tailored to individual patient characteristics, thereby optimizing therapeutic efficiency and minimizing treatment failure.

### 4.1. Genetic and Pharmacogenomic Determinants of GLP-1 Response Variability

Genetic architecture plays a significant role in determining susceptibility to obesity and may also influence variability in response to pharmacological interventions targeting energy balance. Variants in genes involved in appetite regulation and energy homeostasis, including the melanocortin-4 receptor (MC4R), fat mass and obesity-associated gene (FTO), and transcription factor 7-like 2 (TCF7L2), have been associated with differences in body weight regulation, insulin secretion, and metabolic adaptation. In particular, disruptions in the melanocortin pathway have been strongly linked to altered satiety signaling and reduced response to weight loss interventions, highlighting a potential mechanistic intersection with GLP-1-mediated central effects [36].

Genome-wide association studies (GWAS) have identified multiple loci associated with body mass index (BMI) and metabolic traits, reinforcing the polygenic nature of obesity and its treatment response variability [37].

The genetic architecture of obesity, comprehensively reviewed by Goodarzi and colleagues, encompasses both common variants with small effect sizes identified through GWAS and rare monogenic forms that have illuminated critical pathways in energy homeostasis [38].

In addition, pharmacogenomic variability in incretin signaling pathways, including polymorphisms in the GLP-1 receptor gene (GLP1R) and downstream signaling components, has been suggested as a contributor to heterogeneity in glycemic response to GLP-1 RAs, although clinical implementation remains in its early stages [39,40,41].

From a precision nutrition perspective, the interaction between genetic variants and dietary response, the domain of nutrigenomics, is particularly relevant. Gene–diet interaction studies have demonstrated that the effects of specific dietary patterns on weight loss and metabolic outcomes vary according to genetic risk profiles [42,43]. This suggests that individuals with high genetic risk for obesity may derive differential benefits from specific dietary interventions during GLP-1 RA therapy, and that nutrigenomic profiling could potentially guide the personalization of nutritional recommendations to maximize therapeutic outcomes. The concept of phenotype-guided pharmacotherapy, wherein anti-obesity medications are selected based on individual pathophysiological profiles, has shown promise in improving weight loss outcomes and supports the integration of genetic and phenotypic information into treatment algorithms [44,45]. Early studies in adolescent populations suggested that baseline clinical characteristics, including insulin sensitivity and appetite measures, may predict weight loss response to GLP-1 RA therapy, providing a foundation for phenotype-based treatment selection [46]. Similarly, in adults with type 2 diabetes, predictors of GLP-1 RA effectiveness include baseline HbA1c, fasting C-peptide, and duration of diabetes [47].

The variability in weight loss response to GLP-1 RAs shares mechanistic similarities with that observed with SGLT2 inhibitors, including the influence of baseline β-cell function, insulin sensitivity, and compensatory hyperphagia, suggesting common pathways that may inform personalized treatment selection across drug classes [48].

Despite these advances, pharmacogenomic testing cannot yet be routinely recommended for guiding GLP-1 RA therapy in clinical practice. Current evidence derives predominantly from retrospective analyses and association studies, and prospective trials demonstrating that genotype-guided treatment selection improves outcomes are lacking. The clinical implementation of pharmacogenomic information in GLP-1 therapy therefore remains an important research priority rather than an established component of precision obesity pharmacotherapy.

### 4.2. Sex Differences in Metabolic and Neuroendocrine Response to GLP-1 Receptor Agonists

Sex represents a key biological variable influencing metabolic response to GLP-1 receptor agonists, yet it remains underexplored in clinical trial design and therapeutic decision-making. Clinical observations suggest that women often achieve greater relative weight loss compared to men during GLP-1 RA therapy, although variability is influenced by baseline adiposity, hormonal status, menopausal state, and body composition [49].

The biological basis for sex differences in GLP-1 response is multifactorial. Estrogen signaling enhances insulin sensitivity, modulates hypothalamic appetite circuits, and may amplify the anorexigenic effects of GLP-1 receptor activation through interactions with the central melanocortin and serotonergic pathways [50]. Conversely, androgen excess in conditions such as polycystic ovary syndrome (PCOS) is associated with visceral adiposity, insulin resistance, and impaired metabolic flexibility, potentially attenuating GLP-1-mediated improvements in body composition and glycemic control. Men, who typically exhibit greater visceral adipose tissue accumulation and higher rates of metabolic syndrome, may experience different patterns of weight loss, with a potentially higher proportion of lean mass loss during pharmacological weight reduction [51].

Differences in central nervous system sensitivity to satiety and reward signaling pathways further contribute to sex-based variability in treatment outcomes. Neuroimaging studies suggest that women exhibit greater neural responses to food cues in limbic and paralimbic regions, which may be differentially modulated by GLP-1 receptor activation. Additionally, sex differences in gastrointestinal motility and GLP-1-induced gastric emptying delay may contribute to variation in both therapeutic efficacy and gastrointestinal tolerability.

From a nutritional perspective, sex-specific dietary recommendations during GLP-1 therapy may optimize outcomes. Women, who are at higher risk of iron deficiency, osteoporosis, and lean mass loss during weight reduction, may require greater attention to micronutrient adequacy, calcium and vitamin D intake, and protein distribution. Men, who typically have higher absolute lean body mass and protein requirements, may benefit from higher absolute protein intake targets to preserve muscle mass during pharmacologically induced weight loss. The integration of sex-specific nutritional strategies into GLP-1 RA treatment protocols represents an important dimension of precision nutrition that warrants further investigation.

### 4.3. Gut Microbiota Composition and Incretin Sensitivity

The gut microbiota is increasingly recognized as a major regulator of host metabolism and a significant determinant of response to GLP-1 receptor agonists. Microbial metabolites, particularly short-chain fatty acids, stimulate enteroendocrine L-cell secretion of endogenous GLP-1, while gut dysbiosis is associated with obesity, systemic inflammation, and impaired metabolic regulation.

Emerging evidence suggests that interindividual differences in baseline microbiota composition may influence pharmacological response to exogenous GLP-1 receptor agonists [22]. The role of the gut microbiota as a mediator and modulator of GLP-1 RA response, including the potential for microbiome-based stratification and microbiota-targeted nutritional interventions, is comprehensively discussed in Section 7.

### 4.4. Baseline Inflammatory Status and Metabolic Responsiveness

As introduced in Section 3.4, the chronic inflammatory state characteristic of obesity significantly modulates metabolic response to GLP-1 receptor agonists. Elevated circulating levels of inflammatory mediators, including C-reactive protein (CRP), interleukin-6 (IL-6), and tumor necrosis factor-alpha (TNF-α), are associated with impaired insulin signaling, altered adipokine secretion, and dysregulated hypothalamic appetite control. These inflammatory pathways may attenuate GLP-1-mediated improvements in satiety and glucose homeostasis, contributing to variability in therapeutic response [25,33].

As discussed in Section 3.4, inflammation and GLP-1 response are reciprocally linked: while chronic inflammation may blunt the metabolic effects of GLP-1 receptor activation, GLP-1 RAs themselves exert anti-inflammatory effects through the inhibition of NF-κB signaling, reduction in oxidative stress, and modulation of innate immune pathways, including the NLRP3 inflammasome. The net clinical effect may therefore depend on the balance between baseline inflammatory burden and the anti-inflammatory capacity of GLP-1-based therapy.

Importantly, baseline inflammatory status is modifiable through dietary interventions. Anti-inflammatory dietary patterns, characterized by a high intake of polyphenol-rich foods, omega-3 fatty acids, dietary fiber, and minimally processed plant foods, have been shown to reduce circulating inflammatory biomarkers and improve metabolic parameters. This suggests that nutritional strategies aimed at reducing baseline inflammation prior to or concurrently with GLP-1 RA initiation may enhance pharmacological efficacy, particularly in patients with high inflammatory burden. The pharmacometabolic synergy between anti-inflammatory diets and GLP-1 RAs represents a promising avenue for precision nutrition approaches that requires prospective validation.

### 4.5. Sarcopenic Obesity and Body Composition Heterogeneity

Baseline body composition is a critical determinant of response to GLP-1 receptor agonists and a key factor for personalizing nutritional interventions. While pharmacological weight loss is primarily driven by fat mass reduction, a clinically relevant proportion of total weight loss, estimated at approximately 20–30%, may involve fat-free mass, particularly in older adults, individuals with inadequate protein intake, and those with pre-existing deficits in skeletal muscle mass [19]. It should be noted that fat-free mass comprises not only skeletal muscle but also organs, bone, and body water. Changes in hydration status—which may occur during GLP-1 RA therapy due to altered fluid intake or gastrointestinal losses—can influence body-composition measurements obtained through bioelectrical impedance analysis, potentially leading to the overestimation of muscle loss if not appropriately interpreted. Dual-energy X-ray absorptiometry (DXA) provides a more accurate assessment of lean soft tissue mass but may not be feasible in all clinical settings. This phenomenon has important implications for metabolic health, physical function, resting energy expenditure, and long-term weight maintenance [20].

Patients with sarcopenic obesity, characterized by the coexistence of excess adiposity and reduced skeletal muscle mass and function, represent a particularly vulnerable subgroup during GLP-1 RA therapy. In this population, further reduction in lean mass during pharmacological weight loss may exacerbate functional decline, worsen metabolic inflexibility, and increase frailty risk [52]. The pathophysiology involves chronic inflammation-driven anabolic resistance, mitochondrial dysfunction, and physical inactivity, all of which may be amplified during rapid weight loss if nutritional intake is not properly managed.

Real-world data on body composition changes during GLP-1 RA therapy indicate considerable heterogeneity in the proportion of lean mass lost, influenced by baseline muscle mass, dietary protein intake, physical activity levels, and age [53,54]. These findings underscore the importance of baseline body composition assessment and personalized nutritional strategies, particularly adequate protein intake and resistance exercise, to preserve lean mass during GLP-1 therapy.

### 4.6. Metabolic Phenotypes and Therapeutic Heterogeneity

Heterogeneity in GLP-1 receptor agonist response is also strongly influenced by underlying metabolic phenotypes that extend beyond BMI-based obesity classification. Individuals with metabolically unhealthy obesity (MUO) typically exhibit higher insulin resistance, greater visceral adiposity, increased ectopic fat deposition, and elevated inflammatory burden, all of which may blunt pharmacological responsiveness to GLP-1 RAs. In contrast, individuals with metabolically healthy obesity (MHO), characterized by preserved insulin sensitivity, lower visceral fat, and reduced systemic inflammation, may respond more favorably to incretin-based therapies [35].

This heterogeneity supports a shift from BMI-based classification toward a phenotype-driven stratification of obesity, integrating metabolic, inflammatory, and behavioral parameters to predict therapeutic response. The identification of distinct metabolic phenotypes, such as those characterized by predominant central appetite dysregulation versus those driven by peripheral insulin resistance, may enable more precise matching of pharmacological agents to underlying pathophysiology [44]. Predictive factors for weight loss response to GLP-1 RAs identified in real-world studies include baseline HbA1c, fasting glucose, duration of diabetes, concomitant medications, and early weight loss trajectory, providing clinically accessible parameters for the early identification of likely responders and non-responders [49,55].

Real-world evidence from Italian diabetic cohorts has confirmed the heterogeneity in glycemic and weight loss response to weekly semaglutide in routine clinical practice, with baseline HbA1c, diabetes duration, and concomitant insulin therapy emerging as significant predictors of therapeutic outcomes [56]. Additionally, a real-world data analysis identified that concomitant medications, including metformin and SGLT2 inhibitors, were associated with differential weight loss trajectories in patients prescribed semaglutide [57].

The substantial interindividual variability in GLP-1 RA response, driven by genetic, sex-specific, microbial, inflammatory, and body composition-related factors, provides a compelling rationale for the integration of precision nutrition into obesity pharmacotherapy. By accounting for the biological determinants of therapeutic heterogeneity, personalized nutritional strategies can be developed that address the specific needs of distinct patient subgroups: protein-optimized diets for those at risk of lean mass loss, anti-inflammatory dietary patterns for patients with high inflammatory burden, microbiota-targeted interventions for individuals with dysbiosis, and sex-specific nutritional recommendations that account for differences in body composition and metabolic regulation. This stratification approach, informed by baseline phenotyping and early treatment response, represents the foundation of precision pharmacometabolic medicine.

The principal sources of interindividual variability in GLP-1 RA response and the corresponding personalization strategies are summarized in Table 2.

## 5. Nutritional Challenges During GLP-1 Receptor Agonist Therapy

While glucagon-like peptide-1 receptor agonists (GLP-1 RAs) are highly effective in inducing clinically meaningful weight loss and improving cardiometabolic outcomes, their profound effects on appetite suppression, gastric emptying, and energy intake reduction introduce a series of specific nutritional challenges that extend beyond those typically encountered during lifestyle-induced weight loss. The pharmacological suppression of hunger, combined with delayed gastric emptying and altered food preferences, creates a unique metabolic environment in which voluntary energy intake is markedly reduced, but the risk of inadequate protein consumption, micronutrient insufficiency, and loss of lean body mass is amplified. These challenges are particularly relevant in populations with pre-existing nutritional vulnerabilities, including older adults, individuals with sarcopenic obesity, and patients with long-standing obesity-related micronutrient deficiencies, where the consequences of suboptimal nutritional management during pharmacotherapy may compromise long-term functional status and metabolic health. Recognizing and addressing these nutritional risks is therefore an essential component of safe and effective GLP-1 RA therapy.

### 5.1. Reduced Energy and Protein Intake: Risks of Malnutrition and Lean Mass Loss

As introduced in Section 4.5, the preservation of lean body mass during GLP-1 RA-induced weight loss is a central clinical challenge. This section examines the nutritional risks arising from reduced energy and protein intake during therapy. One of the most immediate and clinically significant consequences of GLP-1 receptor agonist therapy is a sustained reduction in total caloric intake, as described in Section 3.1. While this effect is therapeutically desirable for weight loss, it may lead to insufficient energy and protein intake, particularly in patients with pre-existing poor dietary habits, older adults with reduced appetite regulation, or individuals with high metabolic demands. Chronic energy restriction without adequate nutritional compensation predisposes to protein-energy malnutrition, reduced basal metabolic rate, and impaired physical performance. Clinical nutrition research has long established that intentional weight loss interventions must ensure adequate protein intake to preserve metabolic function and prevent nutritional deficiencies, particularly in pharmacologically induced weight loss settings where appetite suppression is externally imposed rather than volitionally controlled [62,63].

The risk of inadequate protein intake is compounded by the observation that patients may spontaneously reduce their consumption of protein-rich foods due to early satiety, altered taste perception, or gastrointestinal discomfort, without proportionally increasing their intake from alternative sources. Patients with sarcopenic obesity—characterized by the coexistence of excess adiposity and reduced skeletal muscle mass and function—represent a particularly vulnerable subgroup, as discussed in Section 3.5. In this population, the proportion of lean mass lost during GLP-1 RA-induced weight reduction may be disproportionately high relative to total weight loss, potentially exacerbating functional decline [52]. Meta-analytical evidence indicates that approximately 20–30% of total weight loss during pharmacological interventions may derive from fat-free mass [64], and real-world studies confirm significant heterogeneity in body composition changes during GLP-1 RA treatment [53,54]. However, the proportion of weight loss attributable to skeletal muscle versus other components of fat-free mass (including body water and organ tissue) cannot be precisely determined from the currently available studies, and changes in hydration status during GLP-1 RA therapy may confound body-composition estimates. These findings underscore the importance of early nutritional intervention and body composition monitoring, which are addressed in the precision nutrition strategies outlined in Section 6.

### 5.2. Micronutrient Deficiencies and Reduced Nutrient Density During Prolonged Appetite Suppression

Sustained appetite suppression induced by GLP-1 receptor agonists may lead to a reduced intake of micronutrient-rich foods, increasing the risk of subclinical or overt deficiencies. This risk arises not only from the quantitative reduction in total food intake, but also from qualitative changes in food selection, as patients may unconsciously reduce their consumption of nutrient-dense foods such as fruits, vegetables, whole grains, and lean proteins in favor of smaller portions of energy-dense, nutrient-poor options that are easier to consume when appetite is suppressed. The resulting decline in dietary micronutrient density and the concentration of vitamins and minerals per unit of energy consumed, raises concerns about long-term nutritional adequacy [65].

Nutrients of particular concern include vitamin B12, iron, vitamin D, calcium, and magnesium. Vitamin B12 deficiency is prevalent in individuals with obesity, partly due to the chronic use of metformin in T2DM, and may be exacerbated during GLP-1 therapy if dietary intake of animal products declines. Iron deficiency is common in premenopausal women with obesity and may worsen if reduced meat consumption is not compensated by alternative iron sources. Vitamin D insufficiency, nearly ubiquitous in obesity due to sequestration in adipose tissue and reduced bioavailability, may be further compounded by reduced dietary intake and limited sun exposure [66]. Calcium and magnesium intakes are frequently suboptimal in individuals with obesity and may decline further during appetite suppression, with potential implications for bone health, muscle function, and metabolic regulation.

Clinical studies on dietary quality during weight loss interventions confirm that micronutrient intake often declines alongside energy intake unless specific attention is paid to nutrient density. In a randomized trial of older overweight and obese adults undergoing diet-induced weight loss with exercise, micronutrient intakes, particularly calcium, vitamin D, and magnesium, fell below the recommended levels in a substantial proportion of participants [67]. Similarly, a study of long-term weight loss maintainers found that while overall dietary quality was superior to that of the general population, micronutrient gaps persisted for several nutrients, highlighting the challenge of achieving nutritional adequacy within a reduced energy intake framework [68].

The clinical consequences of unrecognized micronutrient deficiencies during GLP-1 therapy extend beyond classical deficiency syndromes. However, the prevalence and clinical significance of micronutrient deficiencies specifically attributable to GLP-1 RA therapy (as opposed to pre-existing deficiencies common in obesity) have not been systematically characterized. Routine biochemical screening of all patients receiving GLP-1 RAs is not currently supported by direct evidence. Instead, a risk-stratified approach is recommended. Suboptimal vitamin D and calcium status may accelerate bone loss during weight reduction, increasing fracture risk, a concern already heightened in older adults with obesity undergoing rapid weight loss. Iron deficiency may impair physical performance, cognitive function, and quality of life, undermining the functional benefits of weight loss. Magnesium insufficiency may exacerbate insulin resistance and contribute to muscle cramps, which are already commonly reported during GLP-1 therapy. Patients at higher risk for micronutrient deficiencies—including those with pre-existing deficiencies, previous bariatric surgery, long-standing metformin use (vitamin B12), restrictive dietary patterns, older adults, premenopausal women (iron), individuals with limited sun exposure or darker skin (vitamin D), and those experiencing prolonged severe appetite suppression with markedly reduced dietary variety—should undergo targeted nutritional assessment and biochemical screening before and periodically during GLP-1 RA therapy. In lower-risk individuals, dietary counseling emphasizing nutrient-dense food choices within caloric constraints may suffice, with biochemical screening reserved for those developing symptoms or signs suggestive of deficiency.

### 5.3. Gastrointestinal Adverse Effects and Their Impact on Dietary Tolerance

Gastrointestinal adverse effects are among the most frequently reported side effects of GLP-1 receptor agonists and include nausea, vomiting, early satiety, bloating, constipation, and delayed gastric emptying. These symptoms are particularly pronounced during dose escalation phases and represent a major determinant of treatment discontinuation in clinical practice [16].

Practical clinical guidelines for obesity care in patients with gastrointestinal and liver diseases, jointly developed by ESPEN and UEG, provide a framework for managing gastrointestinal symptoms during weight loss interventions, emphasizing gradual dietary advancement, texture modification, and individualized nutritional support [69].

The underlying mechanism involves both central GLP-1 receptor activation in the area postrema and nucleus tractus solitarius, regions implicated in nausea and emesis, and peripheral effects on gastric accommodation and motility [4].

From a clinical management perspective, gastrointestinal symptoms during GLP-1 RA therapy should be stratified by severity to guide appropriate intervention:

- Mild symptoms (e.g., intermittent nausea not interfering with oral intake, mild constipation manageable with dietary modification, transient early satiety): These can generally be managed with the dietary strategies outlined in Section 5, including smaller meal sizes, reduced dietary fat, slower eating, and adequate hydration. Dose escalation should proceed at the standard or slightly slower pace, and patients should be reassured that symptoms often attenuate with continued treatment.
- Moderate symptoms (e.g., persistent nausea limiting food intake, constipation unresponsive to dietary measures, bothersome bloating): These warrant more structured nutritional intervention, potentially including temporary use of liquid or semi-solid meal replacements, systematic identification and avoidance of trigger foods, and consideration of a slower dose-escalation schedule. Pharmacological management of constipation with fiber supplements or osmotic laxatives may be considered.
- Severe or alarm symptoms (e.g., persistent vomiting with inability to maintain oral hydration, signs of dehydration, severe abdominal pain, suspected gastroparesis, or weight loss exceeding therapeutic targets due to inadequate intake): These require immediate medical review. Temporary dose reduction or treatment interruption should be considered, and alternative etiologies (including gastroparesis unrelated to GLP-1 therapy, pancreatitis, or bowel obstruction) must be excluded. Patients with persistent vomiting should be assessed for electrolyte disturbances and dehydration, and hospital admission may be necessary if oral intake cannot be maintained.

From a nutritional perspective, gastrointestinal symptoms during GLP-1 therapy create a specific set of challenges. Nausea and early satiety may lead to meal skipping, reduced meal size, and aversion to specific foods, particularly those high in fat, which delay gastric emptying and may exacerbate symptoms [16]. Constipation, resulting from delayed gastrointestinal transit, may reduce dietary fiber tolerance and limit the intake of fruits, vegetables, and whole grains that are essential for micronutrient adequacy and gut microbiota health. Chronic nausea and vomiting, when severe, can result in clinically significant reductions in food and fluid intake, leading to dehydration, electrolyte imbalances, and acute nutritional deterioration [21].

Dietary strategies to mitigate these symptoms represent a critical component of nutritional management during GLP-1 therapy and are discussed in detail in Section 6. These include meal size reduction, modification of dietary fat content and type, optimization of meal frequency, texture modification, and avoidance of specific trigger foods identified through individual patient experience. The close relationship between gastrointestinal tolerability and dietary intake underscores the importance of dietitian involvement in GLP-1 RA treatment protocols, particularly during dose titration when gastrointestinal symptoms are most pronounced, and the risk of nutritional inadequacy is highest.

### 5.4. Altered Food Preferences and Dietary Variety

A less recognized but clinically important nutritional consequence of GLP-1 receptor agonist therapy is the alteration of food preferences and reduction in dietary variety. GLP-1 receptor activation modulates hedonic feeding behavior through mesolimbic reward pathways, reducing the appeal of highly palatable, energy-dense foods [29]. While reduced preference for calorie-dense, nutrient-poor foods is therapeutically beneficial, the narrowing of food preferences may also lead to the reduced consumption of certain nutrient-dense foods that patients previously enjoyed, resulting in dietary monotony and reduced overall diet quality.

The loss of dietary variety has implications beyond hedonic satisfaction. Dietary diversity is a well-established predictor of nutritional adequacy, with greater variety associated with a higher likelihood of meeting the micronutrient requirements. If GLP-1 therapy leads to a restricted range of accepted foods, particularly if protein-rich foods or specific food groups are systematically avoided due to altered taste perception or gastrointestinal discomfort, the risk of selective nutritional deficiencies increases. Furthermore, dietary monotony may reduce long-term adherence to nutritional recommendations, as patients may become dissatisfied with a limited dietary repertoire and abandon structured nutritional plans. Nutritional counseling during GLP-1 therapy should therefore actively promote dietary variety within caloric constraints, encouraging patients to rotate protein sources, include a diverse range of vegetables, and experiment with different culinary preparations that accommodate altered taste and satiety while maintaining nutritional adequacy.

The nutritional challenges identified in this section, ranging from protein-energy malnutrition and lean mass loss to micronutrient deficiencies, gastrointestinal intolerability, and reduced dietary variety, highlight the critical need for structured, personalized nutritional interventions during GLP-1 receptor agonist therapy. These challenges cannot be adequately addressed through generic lifestyle counseling; rather, they require precision nutrition strategies that account for individual patient characteristics, including baseline body composition, pre-existing nutritional deficiencies, gastrointestinal tolerability, and metabolic phenotype. The following section examines the evidence-based precision nutrition strategies that can be deployed to address these challenges and optimize therapeutic outcomes during GLP-1 RA therapy.

## 6. Precision Nutrition Strategies to Optimize GLP-1 Receptor Agonist Therapy

The integration of precision nutrition into GLP-1 receptor agonist (GLP-1 RA) therapy represents a fundamental evolution in the management of obesity and type 2 diabetes mellitus, shifting the therapeutic paradigm from pharmacological weight reduction alone to a combined pharmacometabolic approach. While GLP-1 RAs effectively reduce energy intake, improve glycemic control, and modulate cardiometabolic risk, their mechanism of action inevitably induces sustained hypophagia, altered gastrointestinal function, and changes in food preference. These effects create a unique nutritional environment characterized by reduced caloric intake but also increased risk of nutrient imbalance, lean mass loss, and dietary monotony. In this context, precision nutrition is not merely an adjunctive recommendation but a necessary co-therapeutic strategy aimed at preserving metabolic health, optimizing body composition, and enhancing the long-term adherence and sustainability of pharmacological treatment [35]. The following strategies represent evidence-based, clinically actionable precision nutrition interventions designed to address the specific challenges that arise during GLP-1 RA therapy.

### 6.1. Defining Precision Nutrition in the Context of GLP-1 RA Therapy

In this review, ‘precision nutrition’ refers to dietary interventions that are tailored to individual patient characteristics—including metabolic phenotype, body composition, genetic profile, gut microbiota configuration, inflammatory status, and therapeutic response—with the aim of optimizing pharmacological outcomes. This approach is distinguished from routine dietary advice (e.g., adequate hydration, smaller meals, general protein recommendations) by its reliance on patient-level data to inform specific, measurable nutritional targets and by its integration with pharmacotherapy. While some recommendations discussed in this section (e.g., meal size reduction, texture modification) overlap with standard dietary counseling for gastrointestinal tolerability, their application within a precision framework involves systematic assessment, individualized prescription, and ongoing monitoring aligned with pharmacological treatment phases.

### 6.2. High-Protein Nutritional Strategies for Body Composition Preservation

Protein intake represents a central determinant of body composition outcomes during GLP-1 RA-induced weight loss. The suppression of appetite and reduction in food intake induced by GLP-1 signaling frequently results in insufficient protein consumption if dietary quality is not actively preserved. This is particularly relevant because protein is not only a structural macronutrient but also a key regulator of satiety, diet-induced thermogenesis, and skeletal muscle protein synthesis through activation of the mechanistic target of rapamycin (mTOR)-dependent pathways.

During energy restriction, inadequate protein intake contributes to negative nitrogen balance and accelerates the loss of fat-free mass. Meta-analytical evidence in energy-restricted populations suggests that higher protein intakes, typically in the range of 1.2–1.6 g/kg of body weight per day, using ideal body weight or adjusted body weight in individuals with severe obesity to avoid excessive absolute protein loads, significantly attenuate lean mass loss and improve body composition outcomes compared to standard protein diets providing 0.8 g/kg/day [70]. However, this recommendation requires individualization based on several factors: age (older adults may require intakes at the upper end of this range to counteract anabolic resistance); renal function (protein intake should be reduced in patients with chronic kidney disease, typically to 0.6–0.8 g/kg/day, under medical supervision); severity of obesity (protein prescription should be calculated using adjusted rather than actual body weight to avoid excessively high absolute intakes); and physical activity level (individuals engaging in resistance exercise may benefit from intakes at the upper end of the range to support muscle protein synthesis). A randomized controlled trial specifically examining protein intake during weight loss demonstrated that diets providing 1.5 g/kg/day of protein resulted in significantly greater preservation of fat-free mass and enhanced muscle protein synthesis compared to standard protein intakes, despite similar total weight loss [71].

In the context of GLP-1 therapy, protein distribution across meals becomes as equally important as total daily intake. Evenly distributed protein ingestion, approximately 25–30 g of high-quality protein per meal, stimulates repeated muscle protein synthesis responses throughout the day, counteracting anabolic resistance that is particularly prevalent in older adults and individuals with sarcopenic obesity. This has direct implications for sarcopenia prevention and long-term metabolic sustainability during pharmacological weight loss. Practical strategies to achieve adequate protein intake within a reduced energy budget include the incorporation of high-protein foods with low energy density (lean meats, fish, eggs, low-fat dairy, legumes), the use of protein supplements or Foods for Special Medical Purposes (FSMPs) when dietary intake is insufficient, and the strategic timing of protein-rich meals before periods of prolonged fasting to maintain amino acid availability for muscle protein synthesis. The choice of body weight metric for protein calculation (actual vs. ideal vs. adjusted body weight) should be individualized, as actual body weight may overestimate the protein needs in individuals with severe obesity.

Emerging evidence specific to GLP-1 RA therapy supports the importance of structured protein and exercise interventions. A recent review highlighted that resistance exercise combined with adequate protein intake represents the most promising strategy to mitigate lean mass loss during incretin-based weight loss pharmacotherapy, with preliminary data suggesting that patients who engage in regular resistance training during GLP-1 therapy preserve significantly more lean body mass than those who do not [64]. Similarly, a comprehensive review of mitigation strategies for lean mass loss during GLP-1 therapy emphasized the synergistic effects of protein-optimized diets, resistance exercise, and adequate energy availability in preserving muscle mass and metabolic rate [61].

The importance of combining pharmacological therapy with structured exercise is further supported by the finding that liraglutide combined with exercise improved weight loss maintenance and body composition outcomes compared to either intervention alone in adults with obesity [72]. Notably, this evidence derives from general weight-loss populations; studies specifically evaluating protein-optimized diets during GLP-1 RA therapy remain limited [65].

These findings underscore the need for integrated nutritional and physical activity prescriptions as standard components of GLP-1 RA treatment protocols.

### 6.3. Mediterranean Diet as a Metabolic Framework for GLP-1 Therapy

The Mediterranean diet provides a structured dietary pattern that aligns particularly well with the metabolic changes induced by GLP-1 receptor agonists. Beyond its favorable macronutrient composition, rich in monounsaturated fats from extra-virgin olive oil, omega-3 fatty acids from fish, and complex carbohydrates from whole grains and legumes, its clinical relevance in the context of GLP-1 therapy lies in its anti-inflammatory, antioxidant, and microbiota-modulating properties. A high intake of polyphenols, fiber-rich plant foods, and bioactive compounds contributes to improved insulin sensitivity, reduced oxidative stress, and enhanced endothelial function.

The PREDIMED trial, a landmark randomized controlled trial, demonstrated that adherence to the Mediterranean diet significantly reduced the incidence of major cardiovascular events by approximately 30% in high-risk individuals, establishing its role as a cardioprotective dietary pattern [73]. Subsequent meta-analyses have confirmed its association with a reduced incidence of type 2 diabetes, improved glycemic control, and favorable changes in body composition and inflammatory biomarkers [74].

In GLP-1 RA-treated patients, the Mediterranean diet may exert synergistic effects through several mechanisms. The high polyphenol content of the diet amplifies anti-inflammatory pathways already modulated by GLP-1 receptor activation, potentially enhancing the cardiometabolic benefits of pharmacological therapy. The fiber-rich composition promotes short-chain fatty acid (SCFA) production by the gut microbiota, which in turn stimulates endogenous GLP-1 secretion, a positive feedback loop that may augment pharmacological incretin activity [22]. Furthermore, the high palatability and dietary diversity inherent to the Mediterranean diet may improve long-term adherence to nutritional recommendations, which is a critical determinant of sustained pharmacotherapy success in chronic obesity management. Practical implementation during GLP-1 therapy should emphasize the inclusion of extra-virgin olive oil as the primary dietary fat, regular consumption of fatty fish rich in omega-3 fatty acids, abundant intake of vegetables and legumes for fiber and micronutrient density, and a moderate consumption of whole grains and nuts within caloric constraints.

### 6.4. Meal Timing, Circadian Biology, and Chrononutrition

Chrononutrition, the study of how meal timing interacts with circadian biology to influence metabolic health, has emerged as a relevant determinant of therapeutic response in obesity and type 2 diabetes. The circadian system, orchestrated by the suprachiasmatic nucleus of the hypothalamus, regulates rhythmic oscillations in insulin sensitivity, lipid metabolism, glucose tolerance, and gut hormone secretion, including GLP-1. GLP-1 receptor signaling interacts with central and peripheral circadian regulators, suggesting that the timing of food intake may modulate both endogenous incretin tone and the pharmacodynamic response to exogenous GLP-1 receptor agonists.

Time-restricted eating (TRE), a chrononutrition strategy that confines food intake to a consistent daily window of 8–10 h without explicit caloric restriction, has been associated with improvements in insulin sensitivity, blood pressure, and oxidative stress markers even in the absence of significant weight loss. In a seminal study of men with prediabetes, early time-restricted feeding (eating within an 8-h window ending in the early afternoon) improved insulin sensitivity, reduced blood pressure, and decreased oxidative stress compared to eating over 12 h, despite identical caloric intake [75]. These findings have been replicated in studies of individuals with metabolic syndrome, where 10-h time-restricted eating produced clinically meaningful improvements in body weight, blood pressure, and atherogenic lipids [76].

Preclinical models have demonstrated that time-restricted feeding can prevent and reverse metabolic dysfunction induced by diverse nutritional challenges, providing mechanistic support for its application in human obesity management [77]. Fasting regimens and time-restricted feeding also intersect with longevity pathways, suggesting that circadian-aligned eating patterns may confer metabolic benefits beyond weight loss [78].

In the context of GLP-1 RA therapy, chrononutrition strategies may offer several specific advantages. Aligning food intake with the circadian peak of insulin sensitivity, typically in the morning and early afternoon, may optimize postprandial glycemic control and enhance the glucose-dependent effects of GLP-1 receptor activation. Earlier meal timing has also been associated with improved appetite regulation and greater diet-induced thermogenesis, potentially amplifying the weight loss effects of GLP-1 therapy [79].

Systematic reviews of time-restricted eating have confirmed its beneficial effects on body weight, insulin sensitivity, and cardiometabolic risk factors, although the magnitude of benefit varies across populations and study designs [80,81]. A scientific statement from the American Heart Association further emphasized the importance of meal timing and frequency as modifiable determinants of cardiometabolic health [82].

Furthermore, early time-restricted feeding may reduce nocturnal gastroesophageal reflux and improve gastrointestinal tolerability by allowing complete gastric emptying before recumbency, addressing one of the most common adverse effects of GLP-1 RAs.

Practical implementation of chrononutrition during GLP-1 therapy involves recommending consumption of the largest meal earlier in the day, avoiding late-night eating, and maintaining consistent meal timing across days to reinforce circadian entrainment. For patients experiencing pronounced nausea and early satiety, distributing caloric intake across multiple small meals within a defined daytime window may improve both tolerability and nutritional adequacy. The interaction between meal timing, circadian biology, and GLP-1 pharmacology represents an emerging frontier in precision nutrition that warrants further investigation through randomized controlled trials. Time-restricted eating is not appropriate for all patients receiving GLP-1 RA therapy. Contraindications include: type 1 diabetes or insulin-requiring type 2 diabetes with risk of hypoglycemia during fasting periods; history of eating disorders, including binge eating disorder and bulimia nervosa, due to the potential for TRE to exacerbate disordered eating patterns [83]; pregnancy or lactation; advanced chronic kidney disease; and concomitant use of medications requiring food intake for absorption or to prevent gastrointestinal irritation. Older adults at risk of malnutrition or with poor oral intake should avoid TRE, as the restricted eating window may further limit opportunities to meet the protein and energy requirements [84]. In patients with diabetes on sulfonylureas or insulin, TRE should only be implemented with close glucose monitoring and appropriate medication adjustment to prevent hypoglycemia [85].

The TREAT randomized clinical trial demonstrated that 12 weeks of time-restricted eating resulted in modest weight loss compared to consistent meal timing in overweight and obese adults, although the effect was primarily attributable to reduced caloric intake rather than circadian mechanisms alone [86]. A pilot study of 8-h time-restricted feeding in obese adults similarly demonstrated improvements in body weight and metabolic disease risk factors [83].

### 6.5. Fiber Intake and Gut Microbiota Modulation for Incretin Amplification

Dietary fiber plays a mechanistic role in modulating GLP-1 physiology through its effects on gut microbiota composition and short-chain fatty acid (SCFA) production. Fermentation of soluble dietary fiber by colonic bacteria produces acetate, propionate, and butyrate, which act as signaling molecules stimulating enteroendocrine L cells to secrete endogenous GLP-1 via activation of the G protein-coupled receptors GPR41 and GPR43 [34]. While the SCFA–incretin axis is well-established in preclinical models and general populations, direct evidence demonstrating that high-fiber diets enhance the efficacy of exogenous GLP-1 receptor agonists in treated patients is currently lacking. This creates a reciprocal relationship in which diet influences incretin signaling and pharmacological incretin therapy interacts with microbial metabolism.

Experimental and human studies have demonstrated that SCFAs improve insulin sensitivity, reduce hepatic glucose production, enhance gut barrier integrity, and attenuate systemic inflammation, effects that closely parallel the metabolic benefits of GLP-1 receptor activation [22]. In GLP-1 RA-treated individuals, high-fiber diets could theoretically contribute to enhancing endogenous incretin tone, although whether this translates into a clinically meaningful amplification of exogenous GLP-1 receptor agonist effects remains to be demonstrated.

Practical dietary recommendations include the consumption of at least 25–30 g of fiber per day from diverse sources, including vegetables, fruits, legumes, whole grains, nuts, and seeds, to promote microbial diversity and SCFA production. However, fiber introduction should be gradual during GLP-1 therapy, particularly in patients experiencing constipation or bloating, as rapid increases in fiber intake may exacerbate gastrointestinal symptoms. Adequate fluid intake is essential to facilitate fiber transit and prevent obstruction in the context of delayed gastric emptying. For patients unable to meet the fiber requirements through diet alone, soluble fiber supplements such as inulin, psyllium, or partially hydrolyzed guar gum may be considered as adjunctive strategies to support gut microbiota health and incretin amplification [87]. Fiber intake should be individualized based on gastrointestinal tolerability. Patients with pre-existing gastroparesis, severe constipation, or known intestinal strictures should exercise caution with high-fiber diets and avoid insoluble fiber supplements that may exacerbate symptoms or pose a risk of obstruction [88]. In patients with delayed gastric emptying secondary to GLP-1 RA therapy, soluble fiber sources (e.g., psyllium, oat bran, partially hydrolyzed guar gum) are generally better tolerated than insoluble fiber, and adequate fluid intake (≥1.5–2 L/day unless contraindicated) is essential [89]. Fiber intake should be temporarily reduced during acute episodes of severe nausea, vomiting, or abdominal pain, and reintroduced gradually as symptoms resolve [16]. Emerging evidence also suggests that the combination of butyrate-producing fibers with polyphenols may exert synergistic postbiotic effects, enhancing SCFA production and promoting anti-inflammatory and gut barrier-protective actions through complementary microbial metabolic pathways [90]. Similarly, the concept of postbiotic modulation, leveraging bioactive microbial metabolites such as butyrate in combination with dietary fibers and polyphenols, represents a promising strategy to optimize gut microbiota composition and metabolic output during GLP-1 RA therapy [90].

### 6.6. Targeted Supplementation and Management of Micronutrient Adequacy

The profound reduction in energy intake during GLP-1 RA therapy increases the risk of micronutrient insufficiency, particularly in individuals with pre-existing dietary limitations, restrictive eating patterns, or elevated baseline requirements. Micronutrients of particular concern include vitamin D, iron, calcium, magnesium, vitamin B12, and folate. Vitamin D insufficiency, nearly universal in obesity due to sequestration in adipose tissue and reduced bioavailability, plays a role in insulin secretion and immune modulation and may require targeted supplementation to maintain optimal status during weight loss [66]. Iron deficiency is prevalent in premenopausal women with obesity and may worsen if reduced meat consumption during GLP-1 therapy is not compensated by alternative dietary sources or supplementation. Vitamin B12 status requires monitoring in patients with concomitant metformin use, which is common in T2DM populations receiving GLP-1 RAs.

Omega-3 fatty acids, particularly eicosapentaenoic acid (EPA) and docosahexaenoic acid (DHA), exert anti-inflammatory effects through the modulation of eicosanoid synthesis, resolution of inflammation, and incorporation into cell membranes, potentially improving cardiometabolic outcomes when combined with GLP-1 therapy [91]. Given that GLP-1 RAs reduce overall food intake, and fatty fish consumption may decline as part of a reduced total intake, targeted omega-3 supplementation may be warranted in individuals with low baseline intake or elevated cardiovascular risk. Omega-3 PUFAs exert both anti-inflammatory and antioxidant effects that contribute to cardiovascular protection, further supporting their potential role as adjunctive therapy in patients undergoing pharmacological weight loss [92].

Supplementation is not without risk and should not be undertaken indiscriminately. High-dose vitamin D supplementation may cause hypercalcemia, particularly in patients with undiagnosed primary hyperparathyroidism or granulomatous disease [93]. Iron supplementation can cause gastrointestinal irritation, constipation, and nausea—symptoms that may be misattributed to GLP-1 RA therapy—and should be reserved for patients with documented iron deficiency rather than used empirically [94]. Omega-3 fatty acid supplements at high doses (>3 g/day) may increase bleeding risk, particularly in patients on anticoagulant or antiplatelet therapy [95,96]. Calcium supplementation has been associated with an increased risk of renal calculi, and in some observational studies, with cardiovascular events, and should be limited to patients with documented inadequate dietary intake [97]. All supplementation decisions should be guided by biochemical assessment of nutritional status, not by presumptive deficiency based on reduced food intake alone.

Precision supplementation strategies should be guided by baseline nutritional assessment, including dietary intake evaluation, biochemical screening for specific micronutrients, and consideration of individual risk factors such as age, sex, menopausal status, medication use, and comorbid conditions. Routine multivitamin supplementation is not recommended as a universal strategy during GLP-1 therapy, as it may not address specific deficiencies and could provide false reassurance regarding nutritional adequacy. Rather, a targeted approach based on identified or anticipated deficiencies is more aligned with precision nutrition principles and may improve both safety and cost-effectiveness. For patients at increased risk (as defined in Section 5.2), periodic monitoring of nutritional status during long-term GLP-1 therapy is recommended. In the absence of specific risk factors, routine biochemical screening of all GLP-1 RA-treated patients is not currently supported by direct evidence.

### 6.7. Anti-Inflammatory Dietary Patterns and Pharmacometabolic Synergy

The inflammatory pathophysiology of obesity, discussed in Section 3.4 and Section 4.4, may attenuate responsiveness to GLP-1 receptor agonists through the impairment of insulin signaling, disruption of hypothalamic appetite regulation, and promotion of anabolic resistance. Anti-inflammatory dietary patterns, characterized by a high intake of polyphenols, omega-3 fatty acids, dietary fiber, and minimally processed plant foods, and low intake of refined carbohydrates, saturated fats, and ultra-processed products, have been hypothesized to enhance pharmacological efficacy by reducing systemic inflammatory tone and improving insulin sensitivity, although direct evidence of pharmacometabolic synergy in GLP-1 RA-treated patients is lacking. This pharmacometabolic synergy, while mechanistically plausible, has not been directly demonstrated in clinical trials of GLP-1 RA-treated patients and remains a hypothesis requiring prospective validation.

Polyphenols, a diverse class of bioactive compounds found in fruits, vegetables, tea, coffee, red wine, and extra-virgin olive oil, have been shown to modulate NF-κB signaling, reduce oxidative stress, and influence gut microbiota composition toward anti-inflammatory profiles [98]. This hypothesis, while mechanistically plausible, requires confirmation in dedicated clinical trials.

The concept of dietary inflammatory potential has been operationalized through indices such as the Dietary Inflammatory Index, which quantifies the overall inflammatory effect of the diet based on its constituent nutrients and bioactive compounds [99]. Diets with lower inflammatory scores have been associated with reduced circulating inflammatory biomarkers and improved metabolic parameters in observational and interventional studies [100].

In the context of GLP-1 therapy, adoption of a low-inflammatory diet may be particularly beneficial for patients with a high baseline inflammatory burden, such as those with metabolically unhealthy obesity, visceral adiposity, or elevated CRP, who may be at risk of attenuated pharmacological response. Beyond anti-inflammatory effects, specific nutraceutical compounds may also modulate white and brown adipose tissue function, promoting a metabolically favorable shift in adipocyte biology through the regulation of inflammatory signaling pathways [101]. Practical implementation involves increasing the consumption of polyphenol-rich foods (berries, citrus fruits, leafy greens, herbs, spices), ensuring adequate omega-3 fatty acid intake from fatty fish or supplementation, replacing saturated fats with monounsaturated and polyunsaturated fats, reducing the intake of ultra-processed foods, and emphasizing culinary preparations that preserve or enhance the anti-inflammatory properties of foods, such as gentle cooking methods, use of herbs and spices, and incorporation of fermented foods that support gut microbiota health.

### 6.8. Foods for Special Medical Purposes in GLP-1 Receptor Agonist Therapy

Foods for Special Medical Purposes (FSMPs) represent a distinct category of nutritionally formulated products designed for the dietary management of patients with specific medical conditions, including disease-related malnutrition and metabolic disorders. Their role in GLP-1 RA therapy warrants specific consideration, as detailed in the pharmacoeconomic evaluation in Section 10.4.

In the context of GLP-1 receptor agonist therapy, FSMPs may serve several clinically important functions. First, high-protein, nutrient-dense FSMPs formulated for weight management can address the risk of protein-energy malnutrition during prolonged hypophagia, providing a nutritionally complete, calorically defined alternative when voluntary dietary intake is insufficient to meet the protein and micronutrient requirements. This is particularly relevant during dose escalation phases when appetite suppression is most pronounced, and in vulnerable populations such as older adults, individuals with sarcopenic obesity, or patients with pre-existing malnutrition risk [102].

Second, specific FSMP formulations enriched with fiber, omega-3 fatty acids, or specific amino acid profiles may provide adjunctive metabolic benefits that complement the pharmacological effects of GLP-1 RAs. For example, FSMPs containing prebiotic fibers may enhance SCFA production and endogenous GLP-1 secretion [22,34], while those with optimized amino acid profiles may support muscle protein synthesis during weight loss [71]. The use of FSMPs as meal replacements or partial meal substitutes may also simplify dietary management for patients struggling to achieve nutritional adequacy within severe caloric restriction, improving both adherence and nutritional status [67].

From a clinical implementation perspective, FSMPs should be integrated into GLP-1 RA treatment protocols based on individual patient assessment, including baseline nutritional status, dietary intake adequacy, gastrointestinal tolerability, and body composition goals. The choice of specific FSMP formulations should be guided by nutritional composition, clinical evidence supporting their use in metabolic disease, and patient acceptability [102].

The use of FSMPs during GLP-1 RA therapy requires attention to several safety considerations. In patients with diabetes, FSMP formulations with high glycemic index should be avoided or used with caution, and blood glucose monitoring should be intensified when FSMPs are introduced as meal replacements [103]. Patients with renal impairment require FSMPs with appropriately adjusted protein, potassium, and phosphate content [103]. Individuals with lactose intolerance or cow’s milk protein allergy require lactose-free or protein-hydrolyzed formulations. FSMPs should not be used as the sole source of nutrition for prolonged periods without the monitoring of micronutrient status and organ function [102]. Finally, the additional caloric content of FSMPs must be accounted for within the total prescribed energy intake to avoid attenuating the weight loss effects of GLP-1 RA therapy.

While FSMPs incur additional direct costs, their potential to prevent malnutrition, preserve lean body mass, and reduce treatment-related complications may improve the overall cost-effectiveness of GLP-1 RA therapy, as discussed in Section 10.

### 6.9. Integrated Precision Nutrition Protocols

The effective implementation of precision nutrition during GLP-1 receptor agonist therapy requires the integration of the individual strategies described above into coherent, patient-centered protocols that are feasible in clinical practice. Rather than applying all interventions simultaneously, a stepped approach based on individual patient characteristics, therapeutic response, and emerging nutritional challenges is recommended.

Baseline assessment should include comprehensive dietary intake evaluation, body composition measurement, biochemical screening for micronutrient deficiencies and inflammatory biomarkers, and an assessment of gastrointestinal function and dietary preferences. This initial phenotyping allows for the stratification of patients into risk categories and the personalization of nutritional interventions. For example, patients with sarcopenic obesity and low baseline protein intake may be prioritized for intensive protein optimization and resistance exercise prescription. Those with high inflammatory burden may benefit from the early adoption of anti-inflammatory dietary patterns, while patients with prominent gastrointestinal symptoms may require gradual dose escalation combined with targeted dietary modifications to improve tolerability.

Ongoing monitoring during GLP-1 therapy should track dietary intake, body composition changes, gastrointestinal tolerability, physical function, and biochemical markers of nutritional status, with adjustments to nutritional interventions made in response to changing needs over the course of treatment. The integration of digital health tools, including mobile applications for dietary tracking, CGM for glycemic monitoring, and wearable devices for physical activity assessment, may facilitate this iterative personalization process, as discussed in Section 8.

The evidence reviewed in this section demonstrates that precision nutrition strategies, encompassing protein optimization, Mediterranean diet adoption, chrononutrition, fiber and microbiota modulation, targeted supplementation, anti-inflammatory dietary patterns, and judicious use of FSMPs, offer clinically meaningful opportunities to enhance the efficacy, tolerability, and long-term sustainability of GLP-1 receptor agonist therapy. The successful translation of these strategies into clinical practice will require multidisciplinary collaboration, patient education, and the integration of nutritional interventions into standard obesity pharmacotherapy protocols.

The evidence-based precision nutrition strategies for GLP-1 RA therapy are summarized in Table 3, while population-tailored dietary guidance stratified by distinct demographic groups is presented in Table 4.

## 7. Gut Microbiota as a Mediator and Modulator of GLP-1 Receptor Agonist Response

The gut microbiota has emerged as a central regulator of host metabolic homeostasis and a significant determinant of interindividual variability in response to glucagon-like peptide-1 receptor agonists (GLP-1 RAs). Beyond its classical role in energy harvest and nutrient metabolism, the intestinal microbiome actively modulates enteroendocrine signaling, systemic inflammation, and gut–brain communication pathways that are directly involved in appetite regulation and glucose homeostasis. The composition and functional capacity of the gut microbiota influence both endogenous incretin biology and the pharmacological response to exogenous GLP-1 receptor agonists, positioning the microbiome as a key interface between nutrition and pharmacotherapy. This section examines the mechanistic relationships between gut microbiota and GLP-1 RA response, the role of microbial dysbiosis in limiting therapeutic efficacy, and the potential for microbiota-targeted nutritional interventions to enhance pharmacological outcomes.

### 7.1. Responders Versus Non-Responders: Microbiome-Based Stratification of GLP-1 Efficacy

Interindividual variability in response to GLP-1 receptor agonists may be partially explained by differences in gut microbiota composition and functional capacity. The concept of “responders” versus “non-responders” to metabolic interventions has been increasingly linked to specific microbial signatures. Individuals who respond favorably to dietary and pharmacological weight loss interventions tend to exhibit higher baseline microbial diversity and enrichment of taxa associated with improved metabolic health. In contrast, non-responders often display dysbiosis characterized by reduced microbial gene richness and pro-inflammatory microbial profiles. The role of gut microbiota in metabolic disorders extends beyond obesity to encompass type 2 diabetes and metabolic syndrome, with dysbiosis contributing to impaired glucose homeostasis through alterations in energy harvest, inflammation, and gut hormone secretion [58,108].

Landmark studies in nutritional science have demonstrated that baseline microbiome composition can predict personalized metabolic responses to dietary interventions. Zeevi and colleagues showed that a machine learning algorithm integrating microbiome data, blood parameters, and dietary habits accurately predicted individualized postprandial glycemic responses, with microbial features contributing substantially to predictive accuracy [109]. This finding supports the broader concept that gut microbiota composition is a determinant of metabolic responsiveness, extending to pharmacological interventions. In a study of the PREDICT-1 cohort, Berry and colleagues confirmed that postprandial metabolic responses to food are highly individualized and influenced by gut microbiome composition, further supporting the rationale for microbiome-informed precision nutrition [110].

In the specific context of GLP-1 RAs, while direct evidence from microbiome-stratified clinical trials remains limited, the mechanistic basis for microbiome-mediated modulation of incretin response is strong. Microbial metabolites, particularly SCFAs, directly stimulate GLP-1 secretion, modulate insulin sensitivity, and regulate inflammatory tone, all of which influence the net pharmacological effect of GLP-1 receptor activation [34]. It should be emphasized that microbiome-based stratification of GLP-1 RA therapy is an emerging research concept, not a clinically validated tool. Currently, no microbiome biomarkers have been prospectively validated to predict GLP-1 RA response in adequately powered clinical trials. The integration of microbiome profiling into routine clinical decision-making for GLP-1 therapy therefore remains investigational and should not be considered standard practice.

### 7.2. Short-Chain Fatty Acids and the Gut–Incretin Axis

Short-chain fatty acids (SCFAs), primarily acetate, propionate, and butyrate, represent key microbial metabolites with direct relevance to GLP-1 physiology. Produced through the bacterial fermentation of dietary fibers in the colon, SCFAs act as signaling molecules through G protein-coupled receptors GPR41 (FFAR3) and GPR43 (FFAR2), which are abundantly expressed on enteroendocrine L cells, immune cells, and adipocytes. Activation of these receptors by SCFAs stimulates the secretion of endogenous GLP-1 and peptide YY (PYY) from L cells, thereby reinforcing incretin-based metabolic regulation through a diet–microbiota–host signaling axis [22]. Butyrate, in particular, exerts pleiotropic metabolic benefits beyond GLP-1 stimulation. It serves as the primary energy substrate for colonocytes, enhances gut barrier integrity through the upregulation of tight junction proteins, and inhibits histone deacetylases (HDACs), leading to the epigenetic regulation of genes involved in inflammation and metabolism. Propionate contributes to hepatic gluconeogenesis and has been shown to improve insulin sensitivity and reduce hepatic steatosis in experimental models. Acetate, the most abundant SCFA, crosses the blood–brain barrier and may influence central appetite regulation through hypothalamic mechanisms [34].

In the context of GLP-1 RA therapy, the SCFA–incretin axis has dual significance. First, a diet rich in fermentable fiber may enhance endogenous GLP-1 secretion through SCFA-mediated L-cell stimulation, potentially amplifying the therapeutic effects of exogenous GLP-1 receptor agonists and enabling effective treatment at lower pharmacological doses. Second, GLP-1 receptor activation may itself influence gut microbiota composition and SCFA production through changes in gastrointestinal transit time, nutrient delivery to the colon, and bile acid metabolism, creating a bidirectional feedback loop between pharmacological therapy and microbial ecology. Elucidating these interactions is an important frontier in precision pharmacometabolic medicine. At present, the clinical utility of microbiome-based stratification for GLP-1 RA therapy remains investigational.

### 7.3. Dysbiosis, Metabolic Endotoxemia, and Inflammatory Modulation of GLP-1 Responsiveness

Building on the inflammatory mechanisms discussed in Section 3.4 and Section 4.4, this section examines the specific role of gut dysbiosis and metabolic endotoxemia in modulating GLP-1 responsiveness. Gut dysbiosis, characterized by reduced microbial diversity, altered community structure with increased Firmicutes-to-Bacteroidetes ratios, and enrichment of potentially pathogenic bacteria, is strongly associated with obesity, insulin resistance, and systemic low-grade inflammation. Dysbiosis contributes to increased intestinal permeability through the disruption of tight junction protein complexes, facilitating the translocation of bacterial lipopolysaccharides (LPSs) and other pro-inflammatory microbial products into the systemic circulation. This process, termed metabolic endotoxemia, activates Toll-like receptor 4 (TLR4)-mediated innate immune responses, triggering the release of pro-inflammatory cytokines and chemokines that impair insulin signaling in peripheral tissues and disrupt hypothalamic regulation of energy homeostasis [59,60].

The clinical relevance of metabolic endotoxemia in GLP-1 RA therapy lies in its potential to attenuate therapeutic response through several converging mechanisms. Chronic activation of inflammatory cascades, including NF-κB and c-Jun N-terminal kinase (JNK) signaling, impairs insulin receptor substrate (IRS) phosphorylation and downstream metabolic signaling, thereby reducing insulin sensitivity in liver, muscle, and adipose tissue. In the central nervous system, inflammatory mediators can disrupt hypothalamic neuronal networks involved in appetite regulation, potentially blunting the anorexigenic effects of GLP-1 receptor activation [111]. In addition, endotoxin-driven inflammation promotes anabolic resistance in skeletal muscle, which may exacerbate lean mass loss during pharmacological weight reduction [112]. Beyond metabolic endotoxemia, gut dysbiosis has also been implicated in the development of endothelial dysfunction and arterial hypertension through alterations in microbial metabolite production and intestinal barrier integrity, further linking the gut microbiota to the cardiovascular complications of obesity [113].

In addition to endotoxin-mediated pathways, recent evidence suggests that GLP-1 RAs may also reverse leptin resistance through microbiome-dependent mechanisms. Specifically, GLP-1 RA-induced alterations in gut microbial metabolism have been shown to activate the inosine/A2A receptor pathway, contributing to the attenuation of obesity and the reversal of leptin resistance [114].

### 7.4. Nutritional Strategies Targeting the Gut Microbiota During GLP-1 Therapy

The recognition of the gut microbiota as a modulator of GLP-1 response creates opportunities for targeted nutritional interventions aimed at optimizing microbial ecology to enhance pharmacological outcomes. Several evidence-based strategies have been identified that may support or restore a metabolically favorable gut microbiota composition during GLP-1 RA therapy.

Dietary fiber and prebiotics represent the most established microbiota-targeted nutritional intervention. Prebiotic fibers, including inulin-type fructans, galacto-oligosaccharides, and resistant starch, selectively stimulate the growth and metabolic activity of beneficial bacteria, particularly *Bifidobacterium* and *Faecalibacterium* species, enhancing SCFA production and promoting gut barrier integrity [115]. In the context of GLP-1 therapy, prebiotic supplementation may amplify endogenous GLP-1 secretion through SCFA-mediated L-cell stimulation [22,34] while simultaneously improving gastrointestinal tolerability by normalizing bowel habits [87].

Probiotics, live microorganisms that confer health benefits when administered in adequate amounts, have been investigated in obesity and metabolic disease with mixed results. Specific strains, including *Lactobacillus* and *Bifidobacterium* species, *Akkermansia muciniphila*, and *Faecalibacterium prausnitzii*, have shown promise in improving metabolic parameters, reducing inflammation, and enhancing gut barrier function in experimental models and small clinical studies. *A. muciniphila*, in particular, has been associated with improved insulin sensitivity and reduced metabolic endotoxemia in preclinical studies, and pasteurized *A. muciniphila* supplementation improved metabolic parameters in a proof-of-concept human trial. However, the clinical evidence supporting routine probiotic supplementation during GLP-1 therapy remains preliminary, and strain-specific effects, optimal dosing, and long-term safety require further investigation [116].

Routine probiotic supplementation is not currently recommended for all patients receiving GLP-1 RA therapy. Specific populations in whom caution is warranted include immunocompromised individuals, patients with central venous catheters, those with severe acute pancreatitis, and patients with short bowel syndrome or intestinal mucosal barrier disruption, in whom probiotic-associated bacteremia or fungemia has been reported [117,118]. The quality, purity, and viable organism count of commercial probiotic formulations vary considerably, and many products are marketed without rigorous regulatory oversight [119]. Until strain-specific efficacy and safety are demonstrated in prospective trials conducted in GLP-1 RA-treated populations, probiotics should be considered an investigational adjunct rather than a standard component of care.

A precision nutrition approach centered on gut microbiota modulation has been proposed for obesity management, emphasizing the potential of dietary interventions tailored to individual microbiome profiles to enhance weight loss and metabolic outcomes [120].

Polyphenols and other phytochemicals represent an additional microbiota-modulating strategy. Dietary polyphenols are poorly absorbed in the small intestine and reach the colon largely intact, where they undergo microbial metabolism to produce bioactive phenolic acids that exert anti-inflammatory and antioxidant effects while also modulating the microbial community composition [121]. Polyphenol-rich foods, including berries, green tea, coffee, cocoa, and extra-virgin olive oil, have been associated with an increased abundance of beneficial bacteria, reduced endotoxemia, and improved metabolic parameters [122]. The integration of polyphenol-rich foods into the diet during GLP-1 therapy may therefore support both direct anti-inflammatory effects and indirect microbiota-mediated benefits.

Practical implementation of microbiota-targeted nutrition during GLP-1 therapy should be individualized based on baseline microbiome composition (where available), dietary habits, gastrointestinal tolerability, and metabolic phenotype. Gradual increases in dietary fiber intake, incorporation of diverse plant foods to promote microbial diversity, and consideration of targeted prebiotic or probiotic supplementation in high-risk individuals represent clinically feasible strategies [22,34]. The potential for microbiome-based stratification to guide these interventions represents an important direction for future research.

### 7.5. Microbiome Signatures as Potential Predictive Biomarkers of Therapeutic Response: An Emerging Research Frontier

The use of microbiome signatures as predictive biomarkers in clinical practice remains aspirational. Advances in metagenomics and systems biology have enabled the identification of microbial signatures associated with metabolic health and treatment response in exploratory studies, opening the possibility—but not yet the reality—of microbiome-based patient stratification before GLP-1 RA therapy. In a seminal study of the gut microbiome in obesity, Le Chatelier and colleagues demonstrated that individuals with low microbial gene richness exhibited more pronounced obesity-associated metabolic disturbances, including greater adiposity, insulin resistance, and dyslipidemia, compared to those with high gene richness [123]. A metagenome-wide association study in type 2 diabetes identified specific microbial species and functional pathways differentially enriched in diabetic versus non-diabetic individuals, suggesting that microbiome configuration may serve as a biomarker of metabolic disease risk and treatment response [124].

Specific bacterial taxa and functional gene pathways have been proposed as predictive biomarkers of response to dietary and pharmacological interventions. Butyrate-producing bacteria, including *Faecalibacterium prausnitzii*, *Roseburia* species, and *Eubacterium rectale*, are consistently associated with improved metabolic outcomes, while increased abundance of pro-inflammatory taxa such as *Prevotella copri* and certain Bacteroides species has been linked to insulin resistance and poor metabolic flexibility [125]. The ratio of Firmicutes to Bacteroidetes, while oversimplified in earlier literature, remains a gross indicator of microbial community structure that correlates with energy harvest efficiency and adiposity [126].

The integration of microbiome profiling into clinical decision-making for GLP-1 RA therapy is not yet standard practice, but the trajectory of evidence suggests that it may become a valuable component of precision pharmacometabolic medicine. Future studies should investigate whether baseline microbiome signatures can prospectively predict weight loss response, glycemic improvement, and gastrointestinal tolerability during GLP-1 therapy, and whether microbiota-targeted nutritional interventions can convert predicted non-responders into responders. These concepts, while supported by mechanistic evidence, have not yet been demonstrated in randomized controlled trials.

The convergence of microbiome science, precision nutrition, and pharmacotherapy offers an unprecedented opportunity to optimize therapeutic outcomes in obesity and type 2 diabetes. The evidence reviewed in this section establishes the gut microbiota as both a mediator and a modulator of GLP-1 receptor agonist response. Microbial SCFA production enhances endogenous incretin signaling, while dysbiosis and metabolic endotoxemia may attenuate pharmacological efficacy through inflammatory mechanisms. Nutritional strategies targeting the gut microbiota, including dietary fiber, prebiotics, probiotics, and polyphenols, offer promising avenues to optimize microbial ecology and enhance therapeutic response. As metagenomic technologies become more accessible and microbiome-based predictive algorithms are refined, the integration of microbiome profiling into GLP-1 RA treatment protocols may enable a new level of precision in obesity pharmacotherapy.

**Figure 2:**
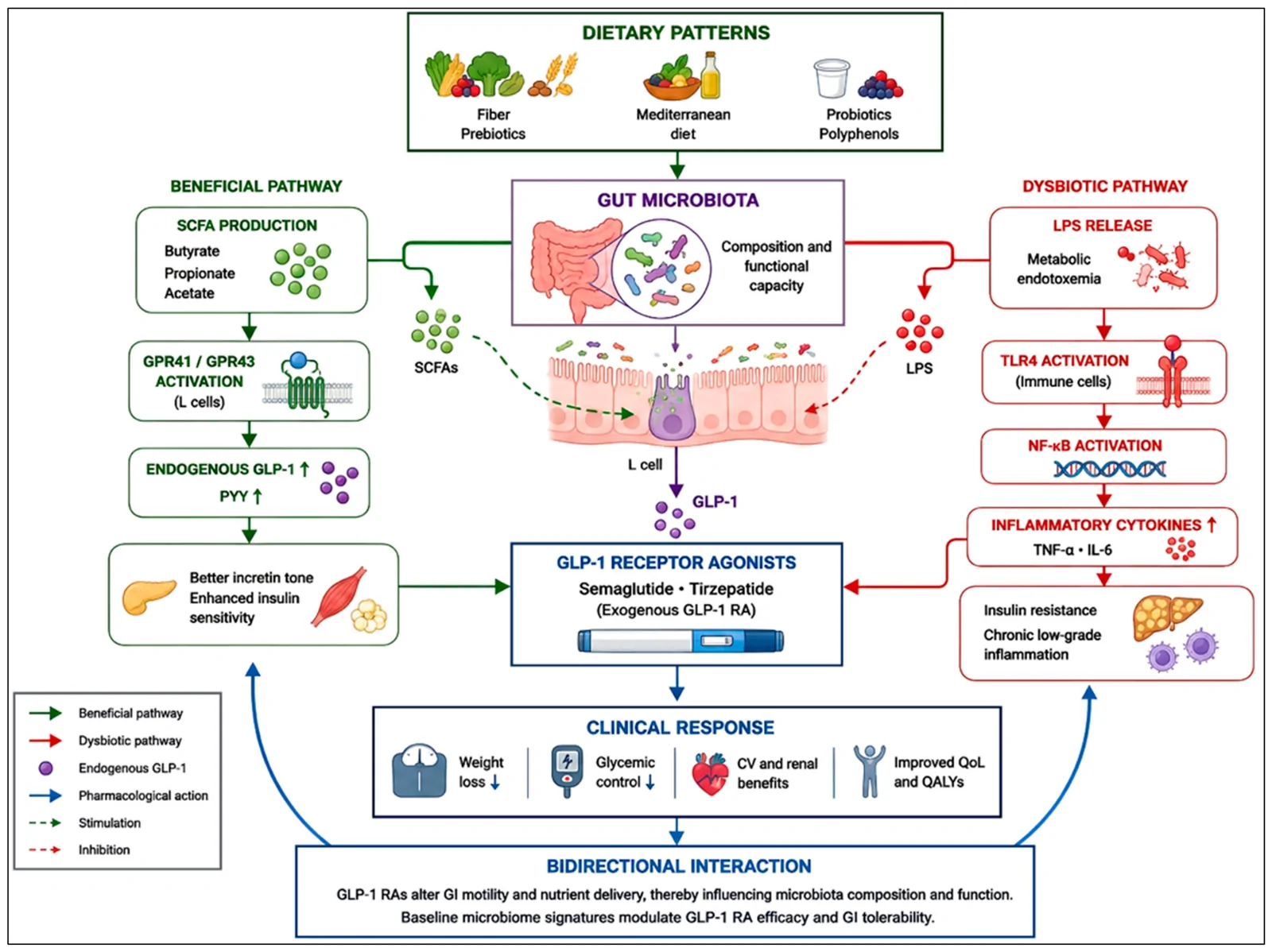
The diet–microbiota–incretin–drug axis. Dietary interventions modulate gut microbiota composition and short-chain fatty acid (SCFA) production. SCFAs stimulate endogenous GLP-1 secretion via GPR41/GPR43 receptors on enteroendocrine L cells, enhancing incretin signaling and insulin sensitivity. Conversely, dysbiosis promotes metabolic endotoxemia through lipopolysaccharide (LPS) translocation, activating TLR4-mediated inflammatory pathways (NF-κB, JNK, TNF-α, IL-6) that may impair metabolic responsiveness. Exogenous GLP-1 receptor agonists (GLP-1 RAs) interact bidirectionally with the gut microbiota, influencing microbial ecology, while their efficacy may be modulated by baseline microbiome composition. Within the Clinical response box, arrows (↓) indicate a decrease in the measured parameter: “Weight loss ↓” indicates body weight reduction, and “Glycemic control ↓” indicates a decrease in blood glucose levels, corresponding to improved glycemic control. Abbreviations: GI, Gastrointestinal; GLP-1, Glucagon-Like Peptide-1; GLP-1 RA, Glucagon-Like Peptide-1 Receptor Agonist; GPR41, G Protein-Coupled Receptor 41; GPR43, G Protein-Coupled Receptor 43; IL-6, Interleukin-6; JNK, c-Jun N-terminal Kinase; LPS, Lipopolysaccharide; NF-κB, Nuclear Factor-kappa B; PYY, Peptide YY; SCFAs, Short-Chain Fatty Acids; TLR4, Toll-Like Receptor 4; TNF-α, Tumor Necrosis Factor-alpha.

At present, the clinical utility of microbiome-based stratification for GLP-1 RA therapy remains investigational. No randomized controlled trial has yet demonstrated that microbiome-guided nutritional or pharmacological interventions improve outcomes in GLP-1 RA-treated patients. The bidirectional interactions between diet, gut microbiota, endogenous incretin signaling, and exogenous GLP-1 receptor agonists are illustrated in Figure 2.

## 8. Biomarkers and Multi-Omics Approaches for Personalized GLP-1 Receptor Agonist Therapy

The increasing recognition of interindividual variability in response to glucagon-like peptide-1 receptor agonists (GLP-1 RAs) has highlighted the need for robust predictive and monitoring tools capable of identifying likely responders and non-responders, guiding dose optimization, and personalizing nutritional interventions. Biomarkers, ranging from routine clinical parameters to advanced metabolomic and genomic signatures, offer the potential to move beyond population-based treatment algorithms toward individualized therapeutic strategies that account for the biological heterogeneity of obesity and type 2 diabetes mellitus. This section examines the role of metabolomics, nutrigenomics, inflammatory biomarkers, and artificial intelligence-driven predictive models in advancing precision pharmacometabolic medicine, with particular attention to their application in GLP-1 RA therapy.

### 8.1. Metabolomics and Metabolic Phenotyping of GLP-1 Response

Metabolomics, the comprehensive analysis of small-molecule metabolites in biological fluids, provides a real-time snapshot of physiological and pathological states, capturing the integrated output of genetic, environmental, dietary, and microbial influences on metabolism. In the context of obesity and type 2 diabetes, metabolomic profiling has been instrumental in identifying metabolic signatures associated with insulin resistance, lipid dysregulation, and energy metabolism, offering insights that are directly relevant to understanding variability in GLP-1 RA response.

Specific circulating metabolites have been consistently linked to insulin resistance and cardiometabolic risk. Branched-chain amino acids (BCAAs), leucine, isoleucine, and valine, and their related catabolites are among the most robustly validated biomarkers of insulin resistance and future diabetes risk. Elevated BCAA levels have been associated with impaired insulin signaling, increased mTOR activation, and disrupted glucose homeostasis, and a BCAA-related metabolic signature has been shown to differentiate obese from lean individuals and to predict the development of type 2 diabetes [127]. A prospective metabolomic analysis further demonstrated that elevated levels of specific amino acids and lipid intermediates predict the future development of diabetes, independent of traditional risk factors [128].

Additional metabolite classes with relevance to GLP-1 RA response include acylcarnitines, markers of incomplete fatty acid oxidation and mitochondrial dysfunction, and specific lipid species such as ceramides and diacylglycerols, which are implicated in hepatic and skeletal muscle insulin resistance [129]. Elevated levels of these metabolites may indicate underlying metabolic inflexibility that could attenuate the insulin-sensitizing and weight loss effects of GLP-1 receptor activation. Conversely, metabolomic signatures characterized by efficient fatty acid oxidation, lower BCAA levels, and a more favorable lipid profile may identify individuals most likely to derive robust metabolic benefits from GLP-1 therapy.

In the context of precision nutrition, metabolomic profiling may guide dietary interventions by identifying specific metabolic pathway perturbations that can be targeted through nutritional strategies. For example, patients with elevated BCAA levels, suggesting impaired BCAA catabolism, may benefit from specific dietary protein modifications or interventions that improve mitochondrial oxidative capacity through exercise and nutrient cofactors [127,128]. Similarly, lipidomic signatures of hepatic steatosis may prompt intensified dietary strategies to reduce hepatic fat, such as reduced fructose and saturated fat intake, which may enhance GLP-1 RA efficacy [130]. The integration of metabolomics into clinical practice remains limited by cost, accessibility, and the need for standardized interpretation algorithms, but its potential to refine therapeutic and nutritional precision is substantial.

Despite their promise, metabolomic approaches to GLP-1 RA therapy personalization face significant barriers to clinical implementation. These include the high cost of metabolomic profiling, limited availability in routine clinical settings, lack of standardized reference ranges for predictive metabolites, and the absence of prospective trials demonstrating that metabolome-guided treatment selection improves outcomes compared to standard care. At present, metabolomics remains a research tool rather than a clinically deployable technology for GLP-1 RA therapy.

### 8.2. Nutrigenomics and Gene–Diet–Drug Interactions

Nutrigenomics explores the bidirectional interactions between genetic variation, dietary exposure, and metabolic response, providing a conceptual and mechanistic bridge between nutrition and pharmacology. Genetic polymorphisms that influence lipid metabolism, insulin secretion, appetite regulation, and inflammatory responses may modify not only dietary response, but also pharmacological efficacy, creating a tripartite gene–diet–drug interaction framework that is central to precision pharmacometabolic medicine.

Variants in genes with established roles in energy homeostasis, including FTO, MC4R, and TCF7L2, have been associated with differences in body weight regulation, appetite control, and glucose metabolism. The FTO locus, the most robustly associated genetic variant with obesity risk, appears to influence energy intake and dietary preference rather than energy expenditure, with functional studies suggesting a role in hypothalamic regulation of appetite and macronutrient selection. MC4R mutations are the most common monogenic cause of obesity and are characterized by hyperphagia and reduced satiety, directly implicating the melanocortin pathway in its pathogenesis. TCF7L2 variants are strongly associated with type 2 diabetes risk and influence insulin secretion, potentially through effects on GLP-1 receptor signaling in pancreatic β-cells [36,37].

The concept that genetic variants may modify both dietary response and drug response has been explored in nutrigenomic studies. Gene–diet interaction analyses have demonstrated that the effects of dietary patterns on weight loss and metabolic outcomes vary according to genetic risk profiles, with individuals at high polygenic risk for obesity potentially deriving greater benefit from specific dietary interventions [42,43]. A study in the Malmö Diet and Cancer cohort demonstrated significant interactions between genetic susceptibility to obesity and dietary fat and carbohydrate intake, with high genetic risk individuals exhibiting greater adiposity when consuming a high-fat diet [131].

Dietary and obesity genetic risk scores have been shown to interact with dietary intake in predicting weight gain, supporting the clinical validity of nutrigenomic approaches to personalized obesity management [132]. A comprehensive scientific statement from the American Heart Association has further outlined the potential of nutrigenomics, microbiome research, and gene–environment interactions to advance personalized approaches to cardiovascular and metabolic disease prevention [133].

The extension of this gene–diet interaction framework to gene–diet–drug interactions in GLP-1 RA therapy is a logical but underexplored frontier. Pharmacogenomic variability in incretin signaling pathways has been documented, with polymorphisms in the GLP-1 receptor gene (GLP1R) associated with differences in glycemic response to GLP-1 RAs [39,40]. It is plausible that individuals with specific GLP1R variants may respond differently not only to pharmacological therapy, but also to dietary interventions that modulate endogenous GLP-1 secretion, creating a complex gene–diet–drug interaction landscape. The clinical implementation of nutrigenomic and pharmacogenomic information in GLP-1 therapy therefore remains aspirational rather than an established component of precision obesity pharmacotherapy. Currently, no guideline-endorsed pharmacogenomic tests exist for GLP-1 RA selection, and the cost-effectiveness of routine genetic testing in this context has not been evaluated. Furthermore, the potential for genetic discrimination and privacy concerns related to genomic data must be addressed before widespread clinical adoption.

### 8.3. Inflammatory and Clinical Biomarkers for Early Response Prediction

Beyond advanced omics technologies, readily accessible clinical and inflammatory biomarkers offer practical tools for predicting and monitoring GLP-1 RA response in routine clinical practice. These biomarkers have the advantage of being widely available, cost-effective, and supported by clinical evidence linking them to therapeutic outcomes.

Systemic inflammatory biomarkers reflect the chronic low-grade inflammation that characterizes obesity and modulates GLP-1 responsiveness. C-reactive protein (CRP), interleukin-6 (IL-6), and tumor necrosis factor-alpha (TNF-α) are elevated in obesity and insulin resistance and have been associated with impaired metabolic response to weight loss interventions. Baseline CRP levels, in particular, have been shown to predict the magnitude of weight loss and glycemic improvement during lifestyle and pharmacological interventions, with higher inflammatory burden generally associated with attenuated response. The lymphocyte-to-monocyte ratio, an integrative marker of systemic inflammation, has been proposed as a prognostic indicator in cardiovascular disease and may have utility in stratifying metabolic risk during GLP-1 therapy [134].

Adipokines, bioactive peptides secreted by adipose tissue, provide additional information on metabolic health and therapeutic responsiveness. Adiponectin, an insulin-sensitizing and anti-inflammatory adipokine, is reduced in obesity and inversely correlated with insulin resistance [135]. Increases in adiponectin levels during weight loss are associated with improvements in insulin sensitivity and may serve as an early marker of favorable metabolic response to GLP-1 therapy. Conversely, leptin, which is elevated in obesity in proportion to fat mass, reflects leptin resistance and may be modulated by GLP-1 receptor activation; a recent meta-analysis of randomized controlled trials demonstrated that GLP-1 RAs significantly reduce circulating leptin levels [136]. Changes in the leptin-to-adiponectin ratio during treatment may provide an integrated measure of adipose tissue function and metabolic improvement.

Early treatment response itself represents one of the most clinically useful predictors of long-term outcomes. Early weight loss, typically defined as weight reduction of ≥5% at 12–16 weeks, has been consistently associated with greater long-term weight loss, improved glycemic control, and higher rates of treatment persistence in GLP-1 RA trials and real-world studies. A retrospective cohort analysis identified early weight loss trajectory, baseline HbA1c, and concomitant medication use as significant predictors of long-term GLP-1 RA response [49]. Similarly, a prospective real-life study identified fasting glucose, HbA1c, and early glycemic improvement as predictors of sustained weight loss during GLP-1 RA treatment [55]. These findings support a practical, clinically implementable approach: patients who fail to achieve meaningful early weight loss or glycemic improvement should be considered for intensified nutritional intervention, dose adjustment, or alternative pharmacological strategies, rather than continuing ineffective therapy.

Biomarkers of body composition and muscle health, including creatinine-based indices, circulating myokines such as myostatin and follistatin, and imaging-derived metrics of muscle mass and quality, are emerging as potentially valuable tools for monitoring lean mass preservation during GLP-1 therapy [137]. Given the clinical importance of preventing excessive lean mass loss, particularly in older adults and individuals with sarcopenic obesity, the development and validation of accessible biomarkers that reflect muscle anabolic–catabolic balance is a research priority.

### 8.4. Artificial Intelligence and Multi-Omics Integration for Precision Pharmacometabolic Medicine

The convergence of multi-omics technologies, clinical data streams, and artificial intelligence (AI) is creating unprecedented opportunities for precision pharmacometabolic medicine. Machine learning algorithms capable of integrating diverse data types, including genomics, metabolomics, microbiome profiles, dietary intake data, continuous glucose monitoring traces, and clinical parameters, can identify non-linear relationships and hidden patterns that are not detectable through conventional statistical approaches, enabling a more accurate prediction of individual therapeutic responses.

The application of AI-based predictive models to GLP-1 receptor agonist therapy, including the potential for machine learning algorithms to integrate multi-omics data for personalized treatment selection, is discussed in Section 9.3.

The translation of AI-driven multi-omics integration into routine clinical care faces significant challenges, including the cost and accessibility of omics technologies, the need for large, diverse training datasets to avoid algorithmic bias, the interpretability of complex machine learning models, and the regulatory and ethical considerations surrounding AI-based clinical decision support [138]. Nonetheless, the trajectory of technological development and the growing evidence base supporting the clinical utility of these approaches suggest that AI-enabled precision pharmacometabolic medicine will become an increasingly important component of obesity and diabetes management in the coming decade.

The biomarkers and omics approaches reviewed in this section collectively support a shift from empirical, trial-and-error GLP-1 RA prescribing toward biomarker-informed, mechanistically rational therapeutic decision-making. Metabolomic signatures of insulin resistance and metabolic inflexibility, nutrigenomic variants influencing drug and dietary response, inflammatory biomarkers reflecting the obesogenic inflammatory milieu, and AI-driven predictive models integrating multi-omics data each contribute to a more comprehensive understanding of interindividual variability in therapeutic response. As these technologies become more accessible and clinically validated, their integration into GLP-1 RA treatment protocols, in conjunction with the precision nutrition strategies outlined in Section 6, will enable increasingly personalized, effective, and efficient obesity pharmacotherapy.

## 9. Digital Health and Personalized Monitoring

The convergence of digital health technologies with precision nutrition and pharmacotherapy represents a transformative opportunity to optimize GLP-1 receptor agonist (GLP-1 RA) treatment through real-time monitoring, data-driven personalization, and enhanced patient engagement. As GLP-1 RAs induce dynamic changes in appetite, glycemic control, gastrointestinal function, and body composition, continuous multimodal monitoring becomes essential to tailor nutritional interventions, detect early signs of intolerability, and predict long-term therapeutic response. In this context, digital health tools, including continuous glucose monitoring, mobile health applications, artificial intelligence-based predictive models, and wearable devices, offer an unprecedented infrastructure for implementing precision pharmacometabolic care.

### 9.1. Continuous Glucose Monitoring and Real-Time Metabolic Feedback

Continuous glucose monitoring (CGM) has emerged as a cornerstone technology for personalized metabolic management, providing real-time data on glycemic excursions, postprandial glucose responses, and glycemic variability. Originally developed for type 1 diabetes management, CGM is increasingly applied in type 2 diabetes, obesity, and even in metabolically healthy individuals to capture individualized responses to dietary intake and pharmacological interventions.

In patients treated with GLP-1 receptor agonists, CGM offers several clinically relevant applications. First, GLP-1 RAs improve glycemic control primarily through glucose-dependent insulin secretion and glucagon suppression, effects that are directly reflected in CGM-derived metrics such as time-in-range (TIR), postprandial glucose excursions, and glycemic variability. Real-time CGM data can therefore provide objective evidence of pharmacodynamic response, helping clinicians distinguish between early responders and those requiring dose adjustment or additional nutritional support [25].

Second, CGM enables real-time feedback on the glycemic impact of specific dietary choices, allowing patients to visualize how different foods, meal compositions, and eating patterns affect their glucose levels during GLP-1 therapy. This biofeedback mechanism reinforces dietary adherence and empowers patients to make informed nutritional decisions aligned with their metabolic phenotype. The predictive value of personalized glycemic responses to meals has been demonstrated in landmark studies showing that individualized postprandial glucose patterns can be accurately predicted by integrating CGM data with microbiome profiles, dietary habits, and clinical parameters [109]. This approach has direct translational potential in GLP-1 RA-treated populations, where interindividual variability in glycemic response remains a major clinical challenge.

Third, CGM may serve as an early warning system for hypoglycemic risk, particularly in patients receiving GLP-1 RAs in combination with sulfonylureas or insulin. Although GLP-1 RAs alone carry a low intrinsic hypoglycemic risk due to their glucose-dependent mechanism of action [4], real-world combination regimens require vigilant glucose monitoring to ensure safety and optimize therapeutic outcomes.

From a precision nutrition perspective, integrating CGM data with digital food logging platforms enables the development of personalized dietary recommendations based on individualized glycemic responses rather than generic caloric prescriptions. This approach aligns with the broader paradigm of precision medicine, where therapeutic and nutritional decisions are guided by real-time biological data rather than population-based algorithms.

### 9.2. Mobile Health Applications and Digital Coaching for Integrated Adherence

Mobile health (mHealth) applications and digital coaching platforms have proliferated as tools to support behavioral change, self-monitoring, and treatment adherence in chronic metabolic diseases. In the context of GLP-1 receptor agonist therapy, mHealth solutions offer a unique opportunity to integrate pharmacological management with nutritional monitoring, physical activity tracking, and gastrointestinal symptom surveillance.

Digital platforms designed for obesity management typically incorporate multiple functionalities, including food logging, weight tracking, physical activity monitoring, medication reminders, and symptom diaries. When applied to GLP-1 RA-treated patients, these tools can capture critical data on dietary intake quality and quantity, meal timing, gastrointestinal tolerability, and changes in appetite and satiety, all of which are directly modulated by GLP-1 receptor activation [29].

Importantly, digital platforms enable the early detection of nutritional inadequacies, including insufficient protein intake, micronutrient gaps, or excessive caloric restriction, that may arise during GLP-1 therapy. By alerting both patients and clinicians to deviations from the optimal nutritional parameters, mHealth tools facilitate timely dietary adjustments and prevent the development of clinically significant deficiencies. This proactive monitoring capability is particularly relevant in older adults, individuals with sarcopenic obesity, and patients with pre-existing malnutrition risk [102].

Digital coaching interventions that combine automated feedback with human support have shown promise in improving weight loss outcomes and treatment adherence in obesity. While large-scale randomized trials specifically evaluating mHealth interventions in GLP-1 RA users remain limited, evidence from diabetes and obesity management broadly supports the efficacy of digital health tools in enhancing metabolic outcomes and patient engagement [139].

The integration of mHealth platforms with electronic health records and clinical decision support systems further extends their utility, enabling seamless communication between patients, dietitians, and prescribing physicians. Such integrated care models are essential for the longitudinal management of obesity, consistent with its recognition as a chronic relapsing disease.

### 9.3. Artificial Intelligence and Predictive Modeling in Pharmacometabolic Response

Artificial intelligence (AI) and machine learning approaches are increasingly being applied to integrate complex, multidimensional datasets, including clinical variables, laboratory parameters, dietary patterns, gut microbiome profiles, and genetic information, to predict individual responses to pharmacological and nutritional interventions. In the context of GLP-1 receptor agonist therapy, AI-based predictive models offer the potential to identify responders and non-responders before treatment initiation, optimize dosing strategies, and personalize nutritional recommendations in real-time.

Machine learning algorithms have demonstrated promising accuracy in predicting metabolic outcomes in obesity interventions. The seminal study by Zeevi and colleagues showed that a machine learning algorithm integrating microbiome data, blood parameters, dietary habits, anthropometrics, and physical activity could accurately predict personalized postprandial glycemic responses to meals, outperforming traditional carbohydrate counting approaches [109]. This proof-of-concept has direct translational relevance to GLP-1 RA therapy, where predicting individualized glycemic and weight loss responses remains a critical unmet need.

Subsequent research has validated the concept that multi-omics integration combined with machine learning can predict dietary responses and metabolic phenotypes with clinically meaningful accuracy [110]. These findings support the development of AI-driven clinical decision support systems that integrate pharmacological, nutritional, and biometric data to guide personalized obesity pharmacotherapy.

In the specific context of GLP-1 RAs, predictive models are being developed to identify baseline characteristics associated with weight loss response, including demographic variables, metabolic biomarkers, and gut microbiota composition [49,55]. Integration of these predictive algorithms into digital health platforms could enable pre-treatment stratification, allowing clinicians to identify patients most likely to benefit from GLP-1-based therapies and those who may require intensified nutritional support or alternative pharmacological strategies.

The convergence of AI, high-performance computing, and digital health has been described as a transformative force in medicine, enabling the transition from population-based guidelines to individualized therapeutic optimization [138]. In obesity pharmacotherapy, this paradigm shift implies that future treatment algorithms will integrate real-time data streams from CGM, wearable devices, microbiome sequencing, and dietary intake logs to continuously adapt pharmacological and nutritional interventions to individual patient needs.

The translation of AI-driven predictive models into routine GLP-1 RA therapy faces substantial barriers. These include the need for large, high-quality training datasets representative of diverse populations to avoid algorithmic bias; the ‘black box’ nature of some machine learning algorithms, which limits clinical interpretability; regulatory uncertainties surrounding AI-based clinical decision support tools; data privacy and security concerns, particularly when integrating genomic, microbiome, and continuous monitoring data; and the absence of prospective trials demonstrating that AI-guided treatment selection improves outcomes or reduces costs compared to standard care. At present, AI-based approaches to GLP-1 RA personalization should be considered investigational.

### 9.4. Wearable Devices and Multimodal Remote Monitoring

Wearable devices, including smartwatches, fitness trackers, and emerging biosensors, provide continuous, passive monitoring of physiological parameters relevant to GLP-1 RA therapy and nutritional status. Physical activity levels, heart rate, heart rate variability, sleep patterns, and estimated energy expenditure are among the metrics routinely captured by consumer-grade wearables, offering a comprehensive picture of lifestyle behaviors that influence metabolic health.

During GLP-1 RA-induced weight loss, wearable-derived data can objectively document changes in physical activity patterns, exercise capacity, and daily energy expenditure, complementing self-reported dietary intake and weight trajectories. Monitoring these parameters is particularly important given concerns about potential reductions in lean body mass and resting energy expenditure during pharmacological weight loss, which may negatively impact long-term metabolic adaptation and weight maintenance [19]. Wearable-derived activity data can inform personalized exercise prescriptions aimed at preserving muscle mass and supporting metabolic health during GLP-1 therapy [64].

Sleep monitoring represents another dimension of wearable utility in GLP-1 RA-treated populations. Circadian disruption and poor sleep quality are associated with impaired glucose tolerance, increased appetite, and altered gut hormone secretion [79]. GLP-1 receptor agonists may influence sleep architecture through central nervous system mechanisms, and monitoring sleep–wake cycles may provide additional insights into treatment response and metabolic adaptation.

Emerging non-invasive biosensors capable of measuring sweat metabolites, interstitial fluid biomarkers, and transdermal analytes hold promise for the continuous monitoring of metabolic parameters without the need for blood sampling [106]. While still primarily in development, such technologies could eventually enable real-time tracking of ketone bodies, lactate, and inflammatory markers, providing a more complete picture of metabolic state during pharmacological weight loss.

The integration of wearable data with CGM, mHealth platforms, and electronic health records creates a comprehensive digital ecosystem for remote patient monitoring [107]. This approach has particular relevance for long-term GLP-1 RA therapy, where sustained adherence, nutritional adequacy, and physical function must be maintained over years of treatment. Remote monitoring also facilitates timely clinical interventions when deviations from the expected therapeutic trajectories are detected, potentially reducing the need for frequent in-person visits and improving healthcare resource utilization, an important consideration in the pharmacoeconomic evaluation of integrated obesity management strategies.

### 9.5. Toward Integrated Digital Precision Pharmacometabolic Platforms

The ultimate goal of digital health integration in GLP-1 receptor agonist therapy is the development of comprehensive precision pharmacometabolic platforms that combine real-time monitoring, predictive analytics, and personalized decision support. Such platforms would integrate data from multiple sources, including CGM, wearable devices, dietary tracking applications, microbiome profiling, and clinical biomarkers, into unified dashboards accessible to patients, clinicians, and allied health professionals.

These integrated systems would enable dynamic adaptation of both pharmacological and nutritional interventions based on real-time physiological data rather than static protocols. For example, the detection of declining protein intake through dietary logging combined with accelerometer-documented reductions in physical activity could trigger automated recommendations for protein supplementation and resistance exercise prescription, preventing excessive lean mass loss. Similarly, CGM data indicating deteriorating glycemic control could prompt early dose adjustment or targeted dietary modifications before clinically significant hyperglycemia develops.

Artificial intelligence algorithms operating on these integrated datasets could continuously refine predictions of long-term outcomes, learning from individual patient trajectories to improve the accuracy of personalized recommendations over time. This closed-loop approach to precision obesity pharmacotherapy represents the convergence of digital health, precision nutrition, and pharmacological innovation envisioned by the broader precision medicine paradigm.

Digital health technologies offer transformative potential for optimizing GLP-1 receptor agonist therapy through personalized monitoring, data-driven nutritional interventions, and predictive analytics. Continuous glucose monitoring provides real-time metabolic feedback that can guide dietary choices and pharmacodynamic assessment. Mobile health applications and digital coaching platforms support integrated adherence by tracking dietary intake, gastrointestinal symptoms, and physical activity. Artificial intelligence and machine learning enable the integration of multidimensional data to predict individual therapeutic responses and personalize nutritional recommendations. Wearable devices provide continuous, passive monitoring of physiological parameters relevant to metabolic health and body composition. The convergence of these technologies into integrated digital precision pharmacometabolic platforms has the potential to enhance therapeutic efficiency, improve patient-centered outcomes, and support the long-term sustainability of GLP-1 RA-based treatment strategies, pending validation in real-world clinical settings. The vision of fully integrated digital precision pharmacometabolic platforms, while compelling, remains aspirational and requires validation in real-world clinical settings before it can be recommended for routine care. The digital health technologies applicable to personalized monitoring during GLP-1 RA therapy are summarized in Table 5.

## 10. Pharmacoeconomic and Clinical Implications

The integration of precision nutrition into GLP-1 receptor agonist (GLP-1 RA) therapy carries profound pharmacoeconomic and clinical implications that extend beyond individual metabolic outcomes to impact healthcare system sustainability. It must be emphasized at the outset that no direct evidence from prospective trials currently demonstrates that adding precision nutrition to GLP-1 RA therapy reduces hospitalizations, improves quality-adjusted life years (QALYs), or enhances cost-effectiveness. The pharmacoeconomic considerations presented in this section are therefore based on indirect modeling, extrapolation from related evidence, and the identification of potential cost-saving mechanisms that require prospective validation. Obesity and type 2 diabetes mellitus (T2DM) impose a substantial economic burden on healthcare systems worldwide, driven by direct medical costs, productivity losses, and the management of obesity-related complications. Within this context, pharmacological weight loss interventions must demonstrate not only clinical efficacy but also economic value, particularly given the high acquisition costs of GLP-1 RAs and the chronic nature of obesity pharmacotherapy. The addition of structured precision nutrition strategies to GLP-1 RA therapy has the potential to enhance cost-effectiveness through multiple mechanisms: improving therapeutic adherence and persistence, reducing treatment-related adverse events and associated healthcare utilization, preserving lean body mass and preventing frailty-related expenditures, preventing micronutrient deficiencies that would otherwise require costly supplementation or hospitalization, and ultimately delivering greater patient-centered outcomes per unit of healthcare expenditure. This section examines the pharmacoeconomic rationale for integrating precision nutrition with GLP-1 receptor agonist therapy, focusing on the economic impact of non-adherence, body composition preservation, adverse event reduction, and the potential for integrated care models to deliver sustainable, cost-effective obesity management. However, direct evidence demonstrating that precision nutrition improves the cost-effectiveness of GLP-1 RA therapy is not yet available, and the economic analyses presented in this section are based on indirect modeling and extrapolation from related evidence.

### 10.1. The Economic Burden of Obesity and the Cost-Effectiveness Imperative

Obesity represents one of the most significant economic challenges facing healthcare systems globally. The direct medical costs of obesity include expenditures for the treatment of obesity itself as well as its numerous complications, type 2 diabetes, cardiovascular disease, chronic kidney disease, metabolic dysfunction-associated steatotic liver disease (MASLD), osteoarthritis, and certain malignancies. Indirect costs encompass productivity losses due to absenteeism, presenteeism, disability, and premature mortality. In the United States, estimates indicate that obesity-related direct medical costs exceed $260 billion annually, with substantially higher per-capita healthcare expenditures among individuals with obesity compared to normal-weight counterparts [140].

The economic rationale for population-level obesity interventions has been comprehensively reviewed, highlighting that the external costs of obesity, including increased healthcare expenditures, reduced workforce productivity, and lost economic output, justify substantial investment in effective prevention and treatment strategies [141].

A comprehensive systematic literature review confirmed the substantial economic burden of obesity and its complications while also demonstrating that clinically meaningful weight loss is associated with significant reductions in healthcare costs, particularly through the mitigation of obesity-related comorbidities [142].

The economic rationale for obesity treatment has been further strengthened by cost-effectiveness analyses comparing pharmacological, surgical, and lifestyle interventions. In an analysis comparing medical therapy, sleeve gastrectomy, and gastric bypass in patients with severe obesity and T2DM, all interventions were found to be cost-effective relative to no treatment, with bariatric surgery offering superior long-term value despite higher initial costs [143]. However, the emergence of highly effective GLP-1 receptor agonists has begun to reshape the cost-effectiveness landscape of obesity pharmacotherapy, with newer agents demonstrating weight loss efficacy approaching that of surgical interventions. A cost-effectiveness analysis of semaglutide 2.4 mg for chronic weight management in Portugal found that the intervention was cost-effective relative to lifestyle modification alone, driven primarily by reductions in obesity-related cardiovascular events and improvements in quality of life [144].

Despite these favorable analyses, the high acquisition cost of GLP-1 RAs remains a significant barrier to widespread adoption and has prompted payers and health technology assessment bodies to scrutinize the real-world value proposition of these therapies. The Institute for Clinical and Economic Review (ICER) has conducted evidence reviews for semaglutide and the dual GIP/GLP-1 receptor agonist tirzepatide in obesity, concluding that while both agents provide substantial clinical benefits, their cost-effectiveness depends critically on long-term adherence, persistence, and the durability of weight loss [145]. In this context, strategies that enhance therapeutic efficiency, including precision nutrition interventions that improve tolerability, adherence, and body composition outcomes, may significantly improve the pharmacoeconomic profile of GLP-1-based obesity pharmacotherapy.

### 10.2. The Cost of Non-Adherence and Therapeutic Discontinuation

One of the most significant threats to the cost-effectiveness of GLP-1 receptor agonist therapy is the high rate of treatment discontinuation observed in real-world clinical practice. While randomized controlled trials report relatively high persistence rates under controlled conditions, real-world evidence consistently demonstrates that a substantial proportion of patients discontinue GLP-1 RA therapy within the first year of treatment, with a recent analysis of commercially insured obese adults without diabetes reporting a one-year persistence rate of only 32.3% and an adherence rate of just 27.2% [146]. Gastrointestinal adverse events, including nausea, vomiting, diarrhea, and constipation, represent the most frequently cited cause of discontinuation, with dose-dependent severity and considerable interindividual variability in tolerability [16].

The pharmacoeconomic consequences of non-adherence and early discontinuation are substantial. Direct costs include the waste of expensive pharmacological agents that are not taken as prescribed or are discontinued before achieving full therapeutic benefit. More importantly, indirect costs arise from the loss of expected clinical benefits: discontinued patients regain weight, experience worsening glycemic control, and lose the cardioprotective and renoprotective effects that would have accrued with sustained therapy. A pooled analysis of SELECT, FLOW, STEP-HFpEF, and STEP-HFpEF DM trials demonstrated the broad cardiorenal benefits of sustained semaglutide therapy, underscoring the clinical and economic importance of maintaining long-term treatment adherence [147,148].

Emerging evidence on strategies for GLP-1 RA discontinuation while maintaining weight loss highlights the potential role of nutritional and behavioral interventions in facilitating dose reduction or temporary treatment interruption without complete weight regain, an approach that could substantially improve the long-term cost-effectiveness of these therapies [149].

As discussed in Section 5.3, gastrointestinal adverse events are the most frequently cited cause of GLP-1 RA discontinuation, and their clinical management through dietary strategies is central to improving treatment persistence. Precision nutrition interventions directly address this adherence gap by mitigating the gastrointestinal adverse effects that drive discontinuation. Dietary modifications, including smaller, more frequent meals; reduction in dietary fat content; optimization of fiber intake; and avoidance of known trigger foods, can substantially reduce nausea, early satiety, and other gastrointestinal symptoms during GLP-1 RA dose escalation. Furthermore, nutritional strategies that improve overall dietary quality and nutrient density within reduced caloric intake can prevent the development of food aversions and dietary monotony that contribute to treatment dissatisfaction. By improving tolerability and treatment satisfaction, precision nutrition may significantly increase the GLP-1 RA persistence rates, thereby enhancing the real-world cost-effectiveness of these therapies.

### 10.3. Pharmacoeconomic Impact of Lean Body Mass Preservation

The potential loss of lean body mass during GLP-1 RA-induced weight loss represents both a clinical concern and a pharmacoeconomic challenge. As discussed in previous sections, a clinically relevant proportion of pharmacologically induced weight loss may involve lean tissue, with significant economic consequences [19]. This phenomenon is particularly pronounced in older adults, individuals with sarcopenic obesity, and those with inadequate dietary protein intake. The clinical aspects of lean mass loss during GLP-1 RA therapy and evidence-based mitigation strategies are examined in detail in Section 4.5 and Section 6.2.

The economic consequences of excessive lean mass loss are mediated primarily through the development or worsening of sarcopenia, frailty, and functional decline. Sarcopenic obesity in older adults is associated with substantially increased risks of falls, fractures, hospitalization, institutionalization, and loss of independence, all of which carry high direct medical costs and long-term care expenditures [52]. Furthermore, reduced skeletal muscle mass lowers resting energy expenditure, potentially contributing to weight regain and the need for repeated or intensified pharmacological interventions over time [20].

Precision nutrition strategies aimed at preserving lean body mass, particularly adequate protein intake (1.2–1.6 g/kg/day), even protein distribution across meals, and concurrent resistance exercise, have demonstrated efficacy in attenuating fat-free mass loss during energy restriction [70,71]. Should these interventions prove similarly effective in GLP-1 RA-treated populations, they could potentially prevent or minimize the loss of metabolically active lean tissue, preserving resting energy expenditure and reducing the risk of weight regain after treatment discontinuation or dose reduction.

From a pharmacoeconomic perspective, the costs of structured nutritional support, including dietary counseling, protein supplementation, and monitoring of body composition, must be weighed against the potentially substantial downstream savings from prevented sarcopenia-related adverse events, maintained functional independence, and improved long-term weight loss durability. While formal cost-effectiveness analyses of protein-optimized nutritional interventions during GLP-1 RA therapy have not yet been published, the existing evidence on the economic burden of sarcopenia and the clinical efficacy of lean mass preservation strategies strongly supports the inclusion of such interventions in value-based obesity pharmacotherapy models.

The pharmacoeconomic case for protein-optimized nutritional interventions during GLP-1 RA therapy, while mechanistically plausible, has not been empirically validated. Formal cost-effectiveness analyses comparing GLP-1 RA therapy with versus without structured nutritional support for lean mass preservation have not been published, and the magnitude of any potential cost savings remains speculative.

### 10.4. Foods for Special Medical Purposes and Integrated Nutritional Support

The Special Issue explicitly calls for contributions addressing the impact of Foods for Special Medical Purposes (FSMPs) and dietary supplements on drug efficacy and treatment outcomes. FSMPs represent a distinct category of nutritionally complete or incomplete formulations designed for the dietary management of patients with specific medical conditions, including disease-related malnutrition and metabolic disorders. In the context of GLP-1 receptor agonist therapy, FSMPs may serve several clinically and economically relevant functions.

First, FSMPs formulated with high protein content and optimized amino acid profiles can address the risk of protein-energy malnutrition during prolonged hypophagia induced by GLP-1 RAs. When voluntary dietary intake is insufficient to meet the protein and micronutrient requirements, a scenario commonly encountered during dose escalation and periods of pronounced appetite suppression, FSMPs provide a nutritionally complete, calorically defined alternative that ensures nutritional adequacy without compromising the caloric deficit necessary for weight loss. This approach aligns with clinical nutrition guidelines for the management of disease-related malnutrition, which emphasize the importance of maintaining nutritional status during catabolic illness states [102,104]. Second, specific FSMP formulations targeting metabolic regulation, such as those enriched with fiber, omega-3 fatty acids, or specific micronutrients, may provide adjunctive metabolic benefits that complement the pharmacological effects of GLP-1 RAs. The anti-inflammatory properties of omega-3 fatty acids, for example, may amplify the anti-inflammatory effects of GLP-1 receptor activation, potentially improving metabolic outcomes in patients with high baseline inflammatory burden [91].

From a health economic perspective, the incorporation of FSMPs into GLP-1 RA treatment protocols may improve cost-effectiveness through several pathways. By preventing the clinical consequences of malnutrition, including impaired immune function, delayed wound healing, muscle wasting, and increased infection risk, FSMPs may reduce hospitalizations and healthcare utilization that would otherwise offset the clinical benefits of pharmacological weight loss [102]. Furthermore, the use of FSMPs as part of a structured nutritional intervention may reduce the need for costly parenteral nutrition or extended hospital stays in patients who develop severe nutritional deficiencies during GLP-1 therapy.

The pharmacoeconomic evaluation of FSMPs in GLP-1 RA-treated populations remains an underexplored area of research and is an important direction for future health technology assessments. Such evaluations should consider not only the direct costs of FSMP provision, but also the potential savings from reduced adverse events, improved treatment adherence, preserved functional status, and enhanced quality of life. As with other aspects of the pharmacoeconomic analysis, direct evidence that FSMPs improve cost-effectiveness in GLP-1 RA-treated patients is not available, and the economic considerations presented here are based on extrapolation from other clinical contexts.

### 10.5. Reduction in Hospitalizations and Complication-Related Costs

The cardiovascular, renal, and metabolic benefits of sustained GLP-1 receptor agonist therapy translate into significant reductions in costly clinical events, including myocardial infarction, stroke, heart failure hospitalization, and progression to end-stage kidney disease. The SELECT trial demonstrated that semaglutide significantly reduced major adverse cardiovascular events in patients with obesity and established cardiovascular disease without diabetes, establishing a new paradigm for cardiovascular risk reduction through obesity pharmacotherapy [14]. Similarly, the FLOW trial demonstrated significant renoprotective effects of semaglutide in patients with type 2 diabetes and chronic kidney disease [6].

These event reductions carry substantial economic implications. Hospitalizations for cardiovascular events, heart failure decompensation, and dialysis initiation are among the most expensive episodes of care in modern healthcare systems. By reducing the incidence of these events, sustained GLP-1 RA therapy generates significant downstream cost savings that partially offset the high acquisition costs of these medications. Should precision nutrition interventions prove effective in enhancing treatment persistence and tolerability, they could potentially amplify these economic benefits by enabling a greater proportion of patients to remain on therapy long enough to accrue cardiovascular and renal protection. However, this remains a theoretical proposition pending direct evidence. Furthermore, obesity-related complications that require hospitalization, including severe infections, venous thromboembolism, and complications of obstructive sleep apnea, may also be reduced through effective weight loss. Nutritional strategies that support sustained weight loss without compromising immune function or nutritional status are therefore integral to maximizing the complication-related cost savings associated with GLP-1 RA therapy.

### 10.6. Patient-Centered Outcomes and Quality-Adjusted Life Years

Beyond clinical events and direct medical costs, the value of integrated nutritional–pharmacological strategies must be assessed in terms of patient-centered outcomes, including health-related quality of life (HRQoL), functional status, and treatment satisfaction. Obesity is associated with significant impairments in HRQoL across the physical, psychological, and social domains, and weight loss interventions have consistently been shown to improve these outcomes.

GLP-1 receptor agonist therapy improves HRQoL through multiple mechanisms, including weight reduction, improved mobility, reduced pain, enhanced self-esteem, and amelioration of obesity-related comorbidities. In the STEP 1-4 trials, semaglutide 2.4 mg significantly improved physical functioning compared to the placebo, with a greater proportion of participants achieving clinically meaningful within-person change in both the IWQOL-Lite-CT Physical Function score (51.8% vs. 28.3% in STEP 1) and the SF-36v2 Physical Functioning score (39.8% vs. 24.1% in STEP 1) [150].

However, treatment-related adverse effects, particularly gastrointestinal symptoms, may partially offset these quality-of-life gains. Precision nutrition interventions that minimize gastrointestinal symptoms while preserving nutritional status may therefore enhance the net HRQoL benefit of GLP-1 therapy, translating into greater quality-adjusted life year (QALY) gains.

In cost-utility analyses, QALYs represent the standard metric for quantifying health benefits, combining both quantity and quality of life into a single measure. Interventions with lower costs per QALY gained are considered more cost-effective. By improving tolerability, adherence, body composition, and long-term metabolic outcomes, precision nutrition strategies have the potential to increase the QALY gains associated with GLP-1 RA therapy while potentially reducing costs from avoided complications and hospitalizations, thereby improving the incremental cost-effectiveness ratio (ICER) of integrated obesity pharmacotherapy. However, no cost-utility analysis has yet evaluated the incremental QALY gain attributable to precision nutrition when added to GLP-1 RA therapy, and the estimates presented in published economic models do not account for structured nutritional interventions.

### 10.7. Toward Sustainable Integrated Care Models

The pharmacoeconomic considerations outlined above support the development of integrated care models that embed precision nutrition within GLP-1 receptor agonist treatment protocols. Such models would involve multidisciplinary teams including physicians, registered dietitians, exercise physiologists, and digital health coaches working collaboratively to optimize pharmacological and nutritional interventions based on individual patient characteristics, preferences, and therapeutic responses.

The economic sustainability of these integrated models depends on several factors: the demonstrated ability of nutritional interventions to improve clinically meaningful outcomes beyond those achievable with pharmacotherapy alone; the costs of delivering nutritional interventions relative to the savings generated through improved adherence, reduced complications, and preserved functional status; and the willingness of payers and health systems to reimburse for nutritional services as integral components of obesity pharmacotherapy rather than optional adjuncts [143]. Current evidence, while not yet definitive, suggests that precision nutrition has the potential to represent a high-value addition to GLP-1 receptor agonist therapy that may enhance therapeutic efficiency and support the long-term sustainability of pharmacological obesity management within resource-constrained healthcare systems. This assessment is based primarily on mechanistic data and indirect economic modeling, and direct cost-effectiveness evidence from prospective trials is currently lacking.

The integration of precision nutrition with GLP-1 receptor agonist therapy represents a promising but still largely unvalidated frontier in metabolic medicine. While the mechanistic rationale is strong and indirect evidence is suggestive, direct clinical trials evaluating combined nutritional–pharmacological strategies are urgently needed to move from hypothesis to evidence-based recommendation. Realizing the potential of precision pharmacometabolic medicine will require sustained investment in interdisciplinary research, pragmatic clinical trials with clearly defined nutritional interventions, health economic evaluations conducted alongside prospective studies, and healthcare system innovation. Until such evidence becomes available, recommendations for precision nutrition during GLP-1 RA therapy should be framed as conditional and based primarily on extrapolation from related evidence rather than on the direct demonstration of efficacy in this specific therapeutic context. The convergence of advances in incretin pharmacology, nutritional biochemistry, microbiome science, multi-omics technologies, and artificial intelligence provides an unprecedented opportunity for research aimed at transforming the management of obesity and type 2 diabetes, with the ultimate goal of improving both individual patient outcomes and the sustainability of healthcare systems. The present review has sought to provide a comprehensive framework for understanding this emerging field, while clearly distinguishing between established evidence, mechanistic hypotheses, and areas requiring prospective validation.

The pharmacoeconomic implications of integrating precision nutrition with GLP-1 RA therapy are summarized in Table 6.

## 11. Limitations

This review has several limitations that should be considered when interpreting its findings. It represents a narrative rather than a systematic review, and the selection and synthesis of evidence may therefore be subject to author bias. The evidence base is heterogeneous, comprising mechanistic studies, observational data, and indirect inferences, with a notable scarcity of randomized controlled trials specifically designed to evaluate precision nutrition interventions during GLP-1 RA therapy. Many of the nutritional strategies discussed are supported by strong mechanistic rationale but await prospective validation in this specific therapeutic context. Pharmacoeconomic analyses are based primarily on indirect modeling, as direct cost-effectiveness data for integrated nutritional–pharmacological strategies remain unavailable. Similarly, the clinical utility of nutrigenomics, metabolomics, artificial intelligence, and wearable-based monitoring in guiding GLP-1 RA therapy remains investigational, with current evidence derived largely from proof-of-concept studies and retrospective analyses rather than prospective interventional trials.

Finally, the field is rapidly evolving, and recommendations may require updating as new evidence emerges. These limitations notwithstanding, the present review provides a timely synthesis of the available evidence, identifies critical knowledge gaps, and offers a framework for future research in precision pharmacometabolic medicine.

## 12. Future Perspectives and Research Priorities

The integration of precision nutrition with GLP-1 receptor agonist therapy represents an evolving field at the intersection of clinical pharmacology, nutritional science, and digital health. While the evidence reviewed in this article supports the rationale and emerging clinical utility of combined nutritional–pharmacological strategies, several knowledge gaps and translational challenges must be addressed to realize the full potential of precision pharmacometabolic medicine in obesity and type 2 diabetes management.

### 12.1. Prospective Clinical Trials of Integrated Nutritional–Pharmacological Interventions

The current evidence base consists primarily of mechanistic studies, observational data, and indirect inferences from trials of dietary interventions or GLP-1 RAs alone. Prospective, randomized controlled trials specifically designed to evaluate the additive or synergistic effects of structured precision nutrition protocols combined with GLP-1 receptor agonist therapy are urgently needed. Such trials should compare GLP-1 RA therapy with standard lifestyle counseling versus GLP-1 RA therapy with intensified, personalized nutritional support, including protein-optimized diets, targeted micronutrient supplementation, microbiota-modulating interventions, and digital health-enhanced monitoring. Key outcomes should include not only weight loss and glycemic control, but also body composition changes (with particular attention to lean body mass preservation), gastrointestinal tolerability, treatment persistence, quality of life, and cost-effectiveness. The STRIDE (Semaglutide Treatment effect in people with obesity: Randomized trial of Integrated Dietary and Exercise intervention) trial concept and similar study designs will be essential to generate the high-quality evidence required to establish integrated nutritional–pharmacological protocols as the standard of care.

### 12.2. Validation of Biomarkers and Algorithms for Patient Stratification

The identification of biomarkers, including metabolomic signatures, microbiome profiles, inflammatory markers, and genetic variants, that predict GLP-1 RA response must progress from discovery and association studies to prospective validation in diverse, real-world populations. Machine learning algorithms integrating multi-omics data with clinical and dietary variables have shown promise in predicting individualized metabolic responses, but their clinical utility in GLP-1 RA therapy remains to be demonstrated in interventional studies. Future research should focus on developing and validating clinically deployable, cost-effective biomarker panels and decision-support algorithms that can be implemented at the point of care to guide GLP-1 RA selection, dosing, and nutritional personalization. The establishment of large, multicenter registries and biobanks linking multi-omics data to longitudinal GLP-1 RA treatment outcomes will be essential to achieve the sample sizes and population diversity required for robust predictive model development.

### 12.3. Mechanistic Elucidation of Diet–Drug–Microbiome Interactions

The bidirectional interactions between dietary composition, gut microbiota ecology, and GLP-1 receptor pharmacology remain incompletely understood. While it is established that microbial short-chain fatty acids stimulate endogenous GLP-1 secretion and that GLP-1 receptor activation influences gastrointestinal transit and nutrient delivery to the colon, the specific microbial taxa, metabolic pathways, and host–microbe signaling mechanisms that mediate these interactions require further elucidation. In particular, the hypothesis that microbiota-targeted nutritional interventions, including prebiotics, specific dietary fibers, and polyphenols, can amplify the therapeutic effects of GLP-1 RAs or convert non-responders into responders requires testing in well-designed human intervention studies with comprehensive metagenomic and metabolomic readouts. The role of postbiotics, bioactive microbial metabolites with established metabolic effects, and next-generation probiotics selected for specific metabolic functions represents a particularly promising area for future investigation.

### 12.4. Development and Evaluation of Foods for Special Medical Purposes for GLP-1 RA Therapy

The Special Issue explicitly calls for contributions addressing the impact of Foods for Special Medical Purposes (FSMPs) on drug efficacy and treatment outcomes, and this represents an important area for future product development and clinical evaluation. Building on the clinical (Section 6.8) and pharmacoeconomic (Section 10.4) considerations discussed above, the development and rigorous evaluation of FSMPs specifically formulated for GLP-1 RA therapy, including high-protein, nutrient-dense formulations for lean mass preservation; fiber-enriched products for microbiota support and glycemic control; and anti-inflammatory formulations enriched with omega-3 fatty acids and polyphenols, could provide standardized, evidence-based nutritional support that complements pharmacological therapy. Future studies should evaluate the clinical efficacy, patient acceptability, and cost-effectiveness of FSMPs as adjuncts to GLP-1 RA therapy, with attention to their impact on body composition, gastrointestinal tolerability, micronutrient status, and long-term metabolic outcomes. The development of personalized FSMP algorithms, matching specific formulations to individual patient phenotypes and nutritional needs, represents a frontier in precision nutrition that aligns closely with the goals of this Special Issue. However, evidence supporting FSMP use specifically during GLP-1 receptor agonist therapy remains limited and largely indirect. Dedicated prospective studies evaluating the clinical efficacy, safety, and cost-effectiveness of FSMPs in this specific therapeutic context are needed before their routine integration into GLP-1 RA treatment protocols can be recommended.

### 12.5. Integration of Digital Health Technologies into Routine Care

The rapid evolution of digital health technologies, including continuous glucose monitors, wearable devices, mobile health applications, and artificial intelligence-driven analytics, creates opportunities for the real-time, data-driven personalization of combined nutritional–pharmacological therapy. Future research should evaluate the clinical effectiveness and cost-effectiveness of integrated digital health platforms that combine GLP-1 RA therapy with personalized nutritional recommendations, remote monitoring of dietary intake and body composition, and automated feedback loops that adjust nutritional prescriptions based on individual responses. The concept of the “digital twin”, a virtual representation of an individual’s metabolic physiology that can be used to simulate responses to pharmacological and nutritional interventions, represents an aspirational goal that could transform precision obesity pharmacotherapy. Pragmatic trials comparing digitally enhanced integrated care with standard care, conducted in real-world settings with diverse populations, will be essential to establish the value proposition of digital precision pharmacometabolic medicine.

### 12.6. Health Economic Evaluations of Integrated Strategies

As healthcare systems increasingly prioritize value-based care and face growing budget constraints, formal health economic evaluations of integrated nutritional–pharmacological strategies will be essential to inform coverage, reimbursement, and clinical guideline development. Cost-effectiveness analyses comparing GLP-1 RA monotherapy with GLP-1 RA plus precision nutrition should incorporate not only direct pharmacological and nutritional intervention costs, but also the potential savings from improved adherence, reduced adverse event-related healthcare utilization, prevention of lean mass loss and associated frailty, and enhanced long-term cardiometabolic outcomes. The pharmacoeconomic framework should also account for patient-centered outcomes, including quality-adjusted life years (QALYs), functional status, and treatment satisfaction. Modeling studies based on data from prospective clinical trials and real-world evidence will be required to project the long-term economic impact of integrated strategies and to identify the patient subgroups for whom these approaches offer the greatest value.

### 12.7. Toward Precision Obesity Medicine: Implementation and Equity

The translation of precision nutrition–GLP-1 RA integration from research settings to routine clinical practice will require addressing significant implementation challenges. These include the training of healthcare professionals in precision nutrition and pharmacometabolic medicine, the development of clinical decision-support tools that integrate multi-omics and digital health data into actionable recommendations, and the establishment of multidisciplinary care models that effectively coordinate physicians, registered dietitians, exercise physiologists, and digital health coaches. Equally important is the need to ensure equitable access to precision pharmacometabolic interventions across diverse populations, avoiding the exacerbation of existing health disparities. The high cost of GLP-1 RAs, advanced biomarker testing, and digital health technologies currently limits access to these interventions for many patients who could potentially benefit. Strategies to reduce costs, develop scalable and affordable precision nutrition tools, and ensure that evidence generation includes diverse racial, ethnic, and socioeconomic populations will be critical to the ethical and equitable implementation of precision obesity medicine.

The integration of precision nutrition with GLP-1 receptor agonist therapy stands at the threshold of a new era in metabolic medicine, one in which pharmacological innovation is matched by equally sophisticated nutritional science and digital health technology, and in which treatment decisions are guided by a deep understanding of individual biology rather than population averages. Realizing this vision will require sustained investment in interdisciplinary research, pragmatic clinical trials, health economic evaluation, and healthcare system innovation. The convergence of advances in incretin pharmacology, nutritional biochemistry, microbiome science, multi-omics technologies, and artificial intelligence provides an unprecedented opportunity to transform the management of obesity and type 2 diabetes, improving both individual patient outcomes and the sustainability of healthcare systems. The present review has sought to provide a comprehensive framework for understanding and advancing this integration, with the hope of stimulating further research, clinical innovation, and evidence-based implementation of precision pharmacometabolic medicine.

## Figures and Tables

**Table 1:** Mechanisms of GLP-1 receptor agonists and nutritional implications.

Pharmacological Mechanism	Primary Site of Action	Clinical Effect	Nutritional Implication	Precision Nutrition Strategy	Evidence Level	References
POMC activation/NPY-AgRP inhibition	Arcuate nucleus, NTS	↓ Appetite, ↑ Satiety	Risk of reduced dietary variety and inadequate protein intake	Small, frequent meals; preserve protein density	B	[28,29]
Delayed gastric emptying	Vagal afferents, ENS	↓ Postprandial glucose excursions, ↑ Satiety	Nausea, early satiety, constipation	Reduce dietary fat; modify food textures; increase meal frequency	A	[4,16]
Glucose-dependent insulin secretion	Pancreatic β-cells	Improved glycemic control, low hypoglycemia risk	Optimizable with low-GI diet	High-fiber diet, slowly absorbed carbohydrates	C	[6,7]
Glucagon suppression	Pancreatic α-cells	↓ Hepatic glucose production	–	–	C	[25,26]
Mesolimbic reward modulation	VTA, nucleus accumbens	↓ Preference for energy-dense foods	Risk of dietary monotony	Promote dietary variety within caloric budget	B	[29,30]
NF-κB and NLRP3 inflammasome inhibition	Peripheral tissues, endothelium	↓ Systemic inflammation	Synergy with anti-inflammatory nutrients	Mediterranean diet, omega-3, polyphenols	C	[14,33]
Microbiota–gut–brain axis modulation	Colon, vagal afferents	↑ Endogenous GLP-1 secretion (SCFA-mediated)	Amplifiable with prebiotic fibers	Fermentable fibers, fiber-enriched FSMPs	C	[22,34]

Abbreviations: ENS, Enteric Nervous System; FSMPs, Foods for Special Medical Purposes; GI, Glycemic Index; NPY, Neuropeptide Y; NTS, Nucleus Tractus Solitarius; POMC, Pro-Opiomelanocortin; SCFAs, Short-Chain Fatty Acids; VTA, Ventral Tegmental Area. Evidence levels: A, direct evidence in GLP-1 RA-treated populations; B, indirect clinical evidence; C, mechanistic/preclinical evidence; D, expert opinion/hypothesis. The only level A evidence in this table is the association between delayed gastric emptying and gastrointestinal symptoms in GLP-1 RA-treated patients; the remaining entries reflect indirect clinical or mechanistic evidence.

**Table 2:** Sources of interindividual variability in GLP-1 receptor agonist response and implications for personalized nutritional strategies.

Determinant	Specific Factors	Impact on GLP-1 RA Response	Personalization Strategy	Evidence Level	References
Genetics	MC4R, FTO, TCF7L2, GLP1R variants	Variability in appetite suppression, insulin secretion	Nutrigenomic profiling; phenotype-guided drug selection	C	[36,37,38,39,40]
Sex	Estrogens, androgens, menopausal status	♀ > ♂ relative weight loss; differential body composition changes	Sex-specific protein targets; attention to iron (♀) and calcium	B	[49,50,51]
Gut microbiota	Microbial diversity, butyrate producers, Firmicutes/Bacteroidetes ratio	Modulation of incretin tone, systemic inflammation	Prebiotics, dietary fiber, targeted probiotics	C	[22,23,34,58]
Inflammatory status	CRP, IL-6, TNF-α, metabolic endotoxemia	↑ Inflammation → ↓ therapeutic response	Anti-inflammatory dietary patterns; omega-3; polyphenols	C	[33,59,60]
Body composition	Baseline lean mass, sarcopenic obesity	↑ Risk of lean mass loss during weight reduction	↑ Protein (1.2–1.6 g/kg/day); resistance exercise	B	[19,20,52,61]
Metabolic phenotype	MUO vs. MHO, insulin sensitivity	MUO: attenuated response	Combined therapy; intensified nutritional support	B	[35,44]

Abbreviations: CRP, C-Reactive Protein; IL-6, Interleukin-6; MHO, Metabolically Healthy Obesity; MUO, Metabolically Unhealthy Obesity; TNF-α, Tumor Necrosis Factor-alpha. Evidence levels: A, direct evidence in GLP-1 RA-treated populations; B, indirect clinical evidence; C, mechanistic/preclinical evidence; D, expert opinion/hypothesis. All entries in this table are classified as B or C, reflecting that the determinants of interindividual variability have been characterized primarily in general populations rather than in studies specifically designed to predict GLP-1 RA response.

**Table 3:** Precision nutrition strategies during GLP-1 receptor agonist therapy.

Strategy	Target	Key Evidence	Practical Recommendation	Safety Considerations	Evidence Level	References
High-protein diet	1.2–1.6 g/kg/day	Attenuation of lean mass loss; preservation of REE	Distribute 25–30 g protein per meal; consider high-protein FSMPs if intake insufficient	Adjust for CKD (0.6–0.8 g/kg/day); use adjusted/ideal BW in severe obesity	B	[64,70,71,72]
Mediterranean diet	Overall dietary pattern	↓ CV events (PREDIMED); ↑ insulin sensitivity; ↓ inflammation	EVOO as primary fat; fatty fish ≥2/week; abundant vegetables, legumes	Generally safe; no specific contraindications	B	[22,73,74]
Time-restricted eating	8–10 h daytime eating window	↑ Insulin sensitivity; ↓ BP	Largest meal earlier in day; avoid late-night eating; consistent meal timing	Avoid in: T1DM/insulin-requiring T2DM, eating disorders, pregnancy, advanced CKD, older adults at malnutrition risk; caution with sulfonylureas/insulin	B	[75,76,80,81,82]
Dietary fiber	25–30 g/day from diverse sources	↑ SCFA production; ↑ endogenous GLP-1; ↓ inflammation	Gradual introduction; adequate hydration; soluble fiber supplements if needed	Caution in gastroparesis/intestinal strictures; prefer soluble fiber; temporarily reduce during severe GI symptoms	C	[16,22,34,89,90]
Omega-3 fatty acids	EPA/DHA intake	↓ Systemic inflammation; improved cardiometabolic outcomes	Supplement if low fish intake or high CV risk	High doses (>3 g/day) may increase bleeding risk, particularly with anticoagulants/antiplatelets	B	[91]
Vitamin D	Maintain optimal serum levels	Insulin secretion, immune modulation	Screening at baseline; supplementation if deficient	Risk of hypercalcemia in hyperparathyroidism/granulomatous disease	B	[66]
FSMPs	Protein, fiber, micronutrient adequacy	Prevention of malnutrition during prolonged hypophagia	High-protein, fiber-enriched formulations; guided by individual assessment	Avoid high-GI formulations in diabetes; adjust protein/K+/phosphate in CKD; account for caloric content; avoid as sole nutrition without monitoring	D	[102,104]
Anti-inflammatory dietary pattern	↓ Dietary Inflammatory Index	↓ CRP, IL-6; ↑ insulin sensitivity	Polyphenol-rich foods; replace SFAs with MUFAs/PUFAs; reduce ultra-processed foods	Generally safe; no specific contraindications	C	[33,98]

Abbreviations: BP, Blood Pressure; CRP, C-Reactive Protein; CV, Cardiovascular; EVOO, Extra-Virgin Olive Oil; FSMPs, Foods for Special Medical Purposes; MUFAs, Monounsaturated Fatty Acids; PUFAs, Polyunsaturated Fatty Acids; REE, Resting Energy Expenditure; SCFAs, Short-Chain Fatty Acids; SFAs, Saturated Fatty Acids. Evidence levels: A, direct evidence in GLP-1 RA-treated populations; B, indirect clinical evidence; C, mechanistic/preclinical evidence; D, expert opinion/hypothesis. The evidence for most nutritional strategies derives from general weight-loss or metabolic disease populations (level B) or mechanistic studies (level C); direct evidence specific to GLP-1 RA-treated patients remains limited.

**Table 4:** Population-specific precision nutrition recommendations during GLP-1 RA therapy.

Population	Protein Target	Key Nutritional Priorities	Special Considerations	Evidence Level	References
Obesity (no T2DM)	1.2–1.6 g/kg IBW/adjBW/day	Lean mass preservation, dietary variety	Monitor micronutrients if severe caloric restriction	B	[19,20,61,64,73,74]
Type 2 diabetes	1.2–1.6 g/kg IBW/adjBW/day (if normal renal function)	Low-GI carbohydrates, consistent meal timing, CGM integration	Assess renal function before high protein intake; align diet with pharmacodynamic profile; TRE only with close glucose monitoring and medication adjustment if on sulfonylureas/insulin	B	[22,25,26,32,85,101]
Older adults (≥65 yr)	≥1.2 g/kg/day; upper end of range	Protein distribution across meals, calcium/vitamin D, resistance exercise	Slow dose escalation to protect nutritional intake; monitor for anabolic resistance; avoid TRE if at risk of malnutrition or with poor oral intake	B	[19,52,61,66,67,84,105]
Sarcopenic obesity	1.2–1.6 g/kg/day + resistance exercise	FSMPs if dietary intake insufficient; body composition monitoring (DXA/BIA)	Gradual dose escalation; avoid rapid weight loss disproportionately affecting muscle	B	[19,20,52,53,61,67]
Chronic kidney disease	0.6–0.8 g/kg/day (non-dialysis CKD stages 3–5); adjust per dialysis status	Monitor potassium, phosphate, fluid intake; avoid renally excreted GLP-1 RAs in advanced CKD	Nephrology collaboration essential; dose adjustment of renally cleared agents; use renal-specific FSMP formulations with adjusted protein/K+/phosphate	B	[5,6,73,106,107]
MASLD	1.2–1.6 g/kg IBW/adjBW/day	Mediterranean diet; limit fructose and saturated fat; target 7–10% weight loss	GLP-1 RAs may provide additive hepatic benefit; consider liver enzyme monitoring	B	[7,76,77,98]
Premenopausal women	1.2–1.6 g/kg IBW/day	Iron and calcium adequacy; attention to bone health during weight loss	Higher risk of iron deficiency with reduced meat intake; monitor menstrual status; iron supplementation only with documented deficiency to avoid unnecessary GI side effects	B	[49,51,68,69,94]

Abbreviations: adjBW, adjusted body weight; BIA, bioelectrical impedance analysis; CGM, continuous glucose monitoring; CKD, chronic kidney disease; DXA, dual-energy X-ray absorptiometry; FSMPs, Foods for Special Medical Purposes; GI, glycemic index; IBW, ideal body weight; MASLD, metabolic dysfunction-associated steatotic liver disease; T2DM, type 2 diabetes mellitus. Evidence levels: A, direct evidence in GLP-1 RA-treated populations; B, indirect clinical evidence; C, mechanistic/preclinical evidence; D, expert opinion/hypothesis. All entries are classified as level B, reflecting that population-specific nutritional recommendations are based on indirect clinical evidence from weight-loss or disease-specific populations; direct evidence specific to GLP-1 RA-treated patients is limited for most nutritional interventions. See Section 6.1 for the definition of precision nutrition and its distinction from routine dietary advice.

**Table 5:** Digital health technologies for personalized monitoring during GLP-1 receptor agonist therapy.

Technology	Parameters Monitored	Role in GLP-1 RA Therapy	Evidence Level	References
Continuous Glucose Monitoring (CGM)	Glycemia, TIR, glycemic variability	Real-time glycemic feedback; guides dietary choices; pharmacodynamic assessment	B	[25,109]
Mobile Health Applications (mHealth)	Dietary intake, weight, GI symptoms, physical activity	Early detection of nutritional inadequacies; integrated adherence support	D	[29,102,139]
Wearable Devices	Physical activity, sleep, HRV, estimated energy expenditure	Personalized exercise prescription; sleep quality monitoring; energy balance tracking	D	[19,64,79]
Artificial Intelligence/Machine Learning	Multi-omics integration, clinical data, dietary patterns	Predictive models for responder/non-responder stratification; personalized recommendations	D	[109,110,138]
Emerging Biosensors	Ketone bodies, lactate, inflammatory markers, sweat metabolites	Non-invasive metabolic monitoring (in development)	D	[49]

Abbreviations: GI, Gastrointestinal; HRV, Heart Rate Variability; TIR, Time-In-Range. Evidence levels: A, direct evidence in GLP-1 RA-treated populations; B, indirect clinical evidence; C, mechanistic/preclinical evidence; D, expert opinion/hypothesis. CGM is supported by indirect clinical evidence (level B); all other technologies are classified as level D, reflecting the absence of randomized controlled trials specifically evaluating their impact on outcomes in GLP-1 RA-treated populations.

**Table 6:** Pharmacoeconomic implications of integrating precision nutrition with GLP-1 receptor agonist therapy.

Clinical Problem	Economic and Clinical Cost	Precision Nutrition Intervention	Potential Savings	Evidence Level	References
Treatment discontinuation/non-adherence	Wasted drug costs; lost CV and renal benefits	Dietary strategies for GI tolerability; nutritional counseling	↑ Persistence → ↑ clinical outcomes → ↓ CV events and hospitalizations	D	[16,147,148,149]
Lean body mass loss/sarcopenia	↑ Falls, fractures, institutionalization, long-term care costs	↑ Protein (1.2–1.6 g/kg/day) + resistance exercise	↓ Long-term care expenditures; maintained functional independence	D	[19,20,52,70,71]
Micronutrient deficiencies	Anemia, bone loss, impaired immune function, infections	Targeted supplementation; FSMPs	↓ Hospitalizations for deficiency-related complications	D	[65,66,67,68]
GI adverse events	Treatment dropout; unplanned medical visits; switch costs	Meal size reduction; fat modification; texture adaptation	↑ Persistence; ↓ costs of alternative therapies	D	[16,69]
Loss of cardiovascular and renal protective benefits	MI, stroke, heart failure, dialysis (high-cost events)	Adherence support through nutrition and digital health	↓ CV and renal hospitalizations (SELECT, FLOW)	D	[14,147,148]
Reduced quality of life	Impaired HRQoL in obesity	Improved tolerability and metabolic outcomes	↑ QALY; improved ICER of integrated therapy	D	[144,145]

Abbreviations: CV, Cardiovascular; FSMPs, Foods for Special Medical Purposes; GI, Gastrointestinal; HRQoL, Health-Related Quality of Life; ICER, Incremental Cost-Effectiveness Ratio; MI, Myocardial Infarction; QALY, Quality-Adjusted Life Year. Evidence levels: A, direct evidence in GLP-1 RA-treated populations; B, indirect clinical evidence; C, mechanistic/preclinical evidence; D, expert opinion/hypothesis. All entries in this table are classified as evidence level D, reflecting that no prospective health economic evaluations have directly demonstrated that adding precision nutrition to GLP-1 RA therapy reduces costs or improves cost-effectiveness. The ‘Potential savings’ represent hypothesized benefits based on indirect modeling and extrapolation from related clinical contexts.

## Data Availability

No new data were created or analyzed in this study. Data sharing is not applicable to this article.

## Abbreviations

AI	Artificial Intelligence
BCAA	Branched-Chain Amino Acid
BMI	Body Mass Index
BP	Blood Pressure
cAMP	Cyclic Adenosine Monophosphate
CGM	Continuous Glucose Monitoring
CRP	C-Reactive Protein
CV	Cardiovascular
DHA	Docosahexaenoic Acid
ENS	Enteric Nervous System
EPA	Eicosapentaenoic Acid
Epac2	Exchange Protein directly Activated by cAMP 2
EVOO	Extra-Virgin Olive Oil
FSMPs	Foods for Special Medical Purposes
FTO	Fat Mass and Obesity-Associated Gene
GI	Gastrointestinal
GLP-1	Glucagon-Like Peptide-1
GLP-1 RA	Glucagon-Like Peptide-1 Receptor Agonist
GLP1R	Glucagon-Like Peptide-1 Receptor gene
GPR	G Protein-Coupled Receptor
GWAS	Genome-Wide Association Study
HbA1c	Glycated Hemoglobin
HDAC	Histone Deacetylase
HRQoL	Health-Related Quality of Life
HRV	Heart Rate Variability
ICER	Incremental Cost-Effectiveness Ratio
IL-6	Interleukin-6
JNK	c-Jun N-terminal Kinase
LPS	Lipopolysaccharide
MASLD	Metabolic Dysfunction-Associated Steatotic Liver Disease
MC4R	Melanocortin-4 Receptor
MHO	Metabolically Healthy Obesity
mHealth	Mobile Health
ML	Machine Learning
mTOR	Mechanistic Target of Rapamycin
MUFAs	Monounsaturated Fatty Acids
MUO	Metabolically Unhealthy Obesity
NF-κB	Nuclear Factor-kappa B
NPY	Neuropeptide Y
NTS	Nucleus Tractus Solitarius
PKA	Protein Kinase A
POMC	Pro-Opiomelanocortin
PUFAs	Polyunsaturated Fatty Acids
PYY	Peptide YY
QALY	Quality-Adjusted Life Year
QoL	Quality of Life
REE	Resting Energy Expenditure
SCFAs	Short-Chain Fatty Acids
SFAs	Saturated Fatty Acids
T2DM	Type 2 Diabetes Mellitus
TCF7L2	Transcription Factor 7-Like 2
TIR	Time-In-Range
TLR4	Toll-Like Receptor 4
TNF-α	Tumor Necrosis Factor-alpha
TRE	Time-Restricted Eating
VTA	Ventral Tegmental Area
